# Supplementary material for: Genome-wide associations of aortic distensibility suggest causality for aortic aneurysms and brain white matter hyperintensities
Source: Nat Commun. 2022 Aug 3;13:4505. doi: 10.1038/s41467-022-32219-x (PMC9349177; doi:10.1038/s41467-022-32219-x)
Supplement: Supplementary file 1 — Supplementary Information [file 41467_2022_32219_MOESM1_ESM.pdf]

Supplementary Figure 1

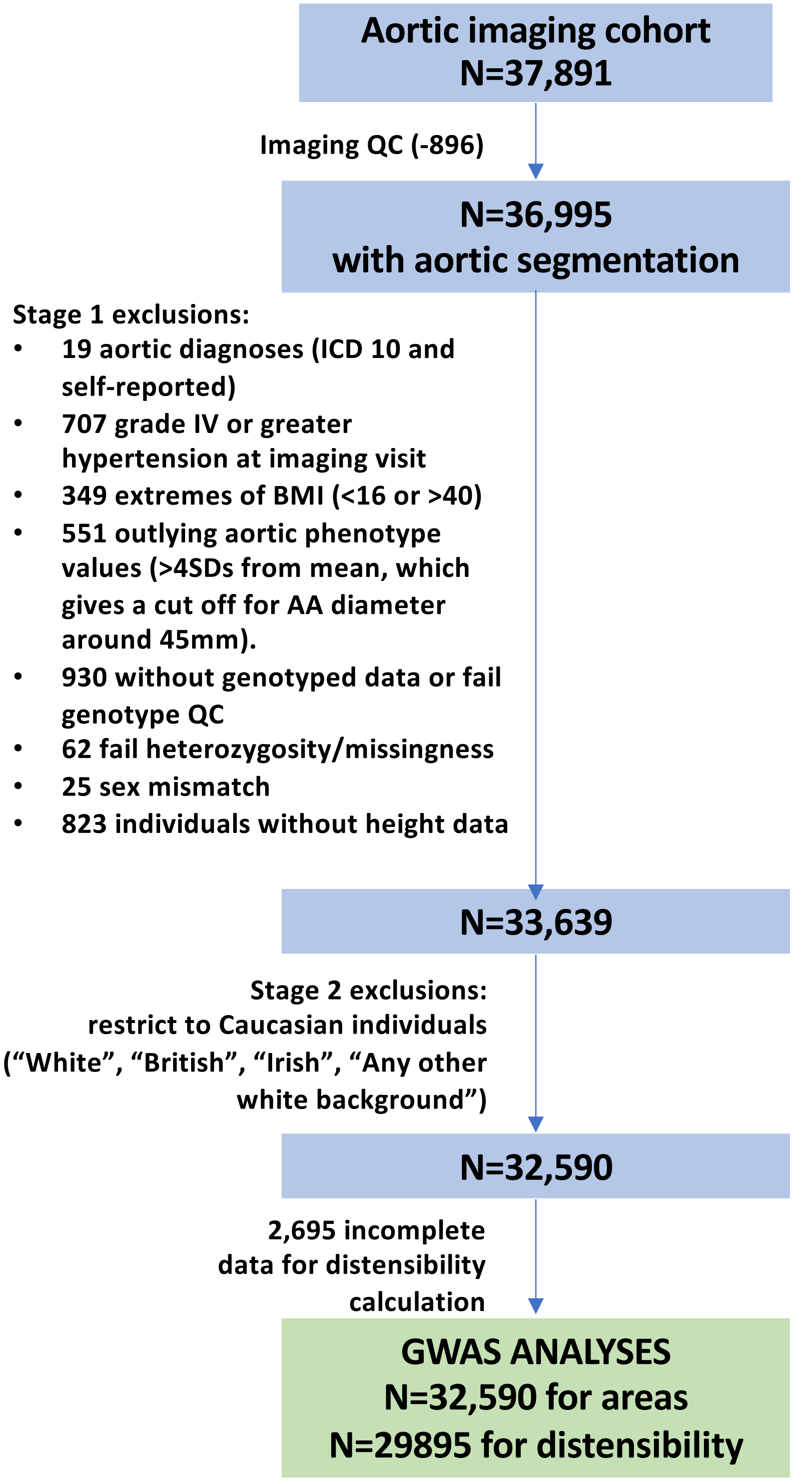

Supplementary Figure 1: Details of exclusions

Supplementary Figure 2

a

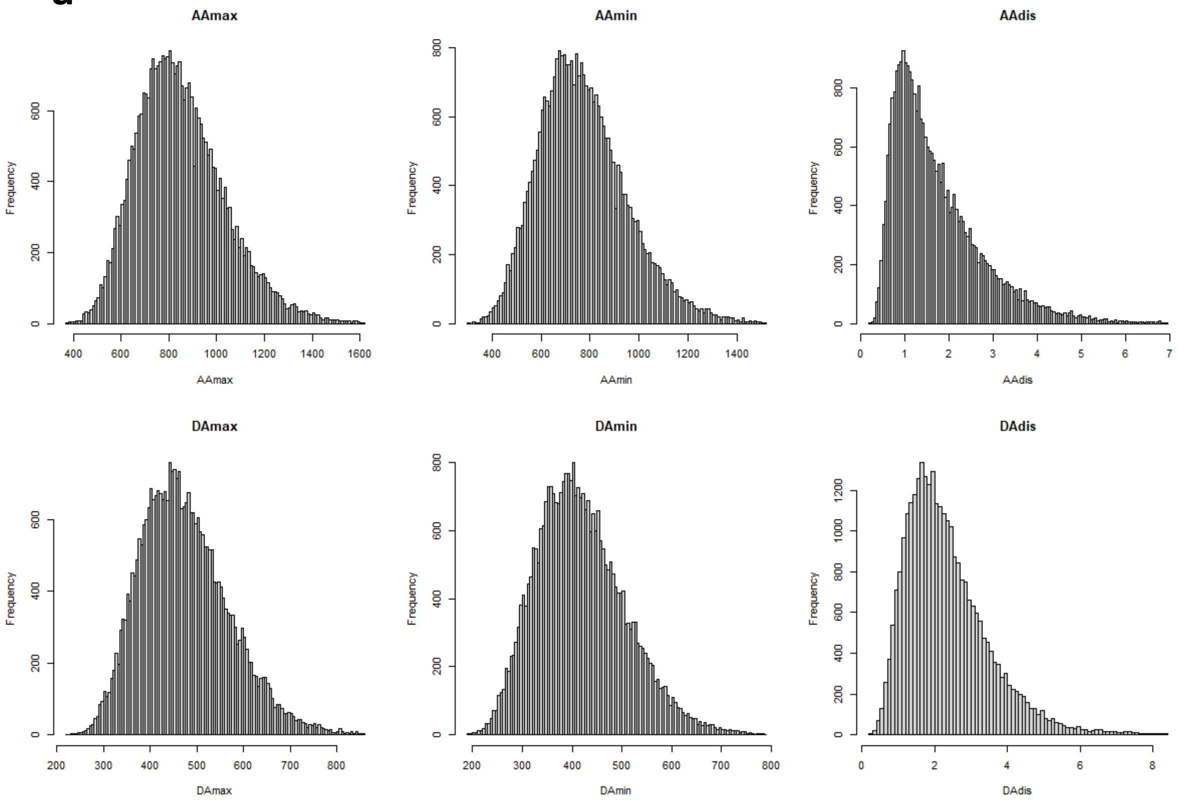

b

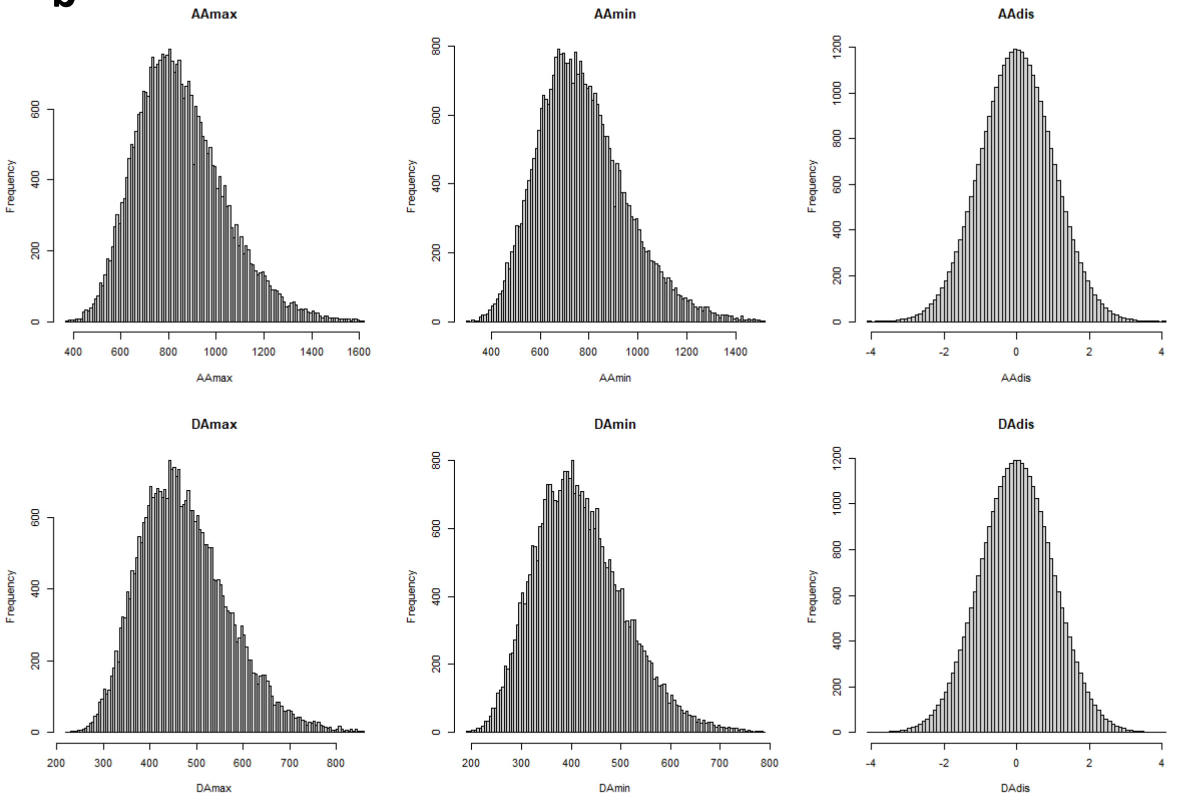

Supplementary Figure 2: Distribution of image phenotype measures: a) Original data and **b**) after rank normalisation for distensibility measures has been applied.

Supplementary Figure 2

c

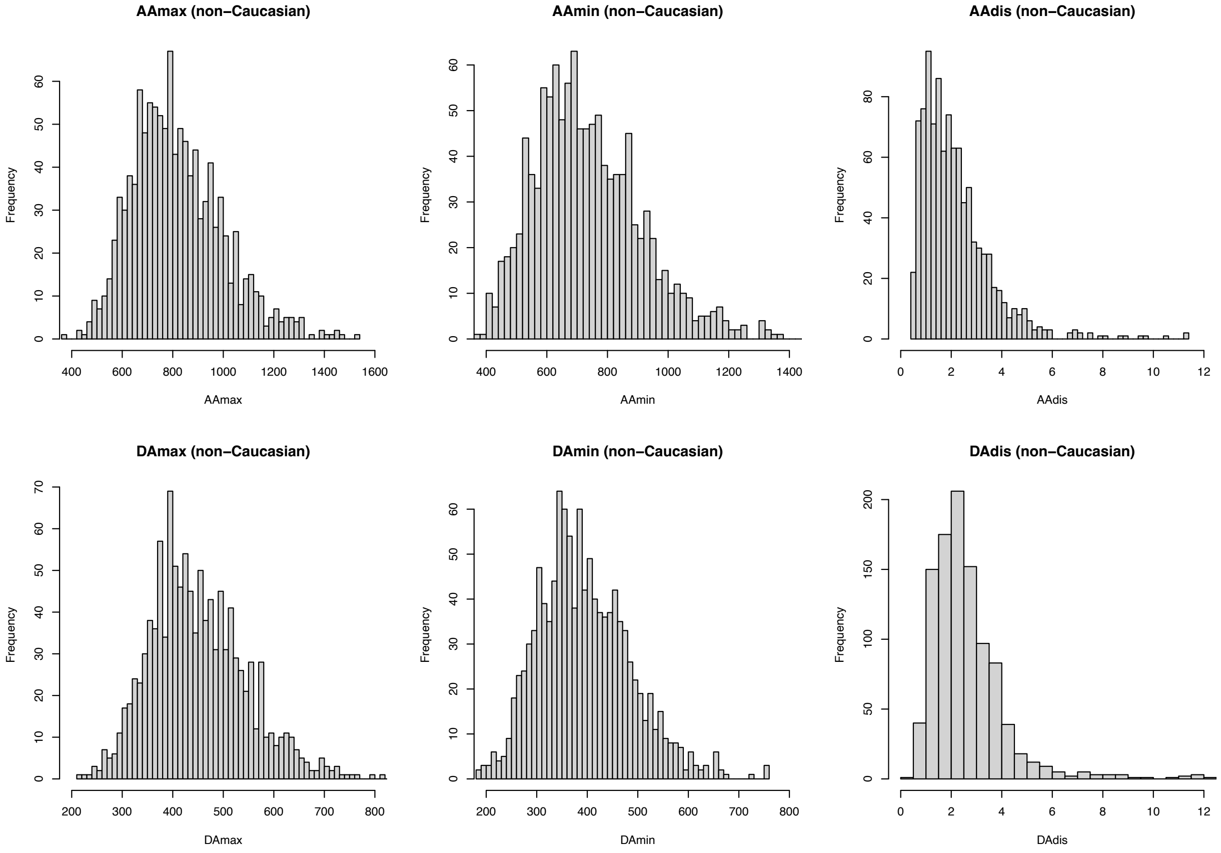

Supplementary Figure 2, panel c) Distribution of image phenotype measures in non-Caucasian individuals (excluded from GWAS analysis).

Supplementary Figure 3

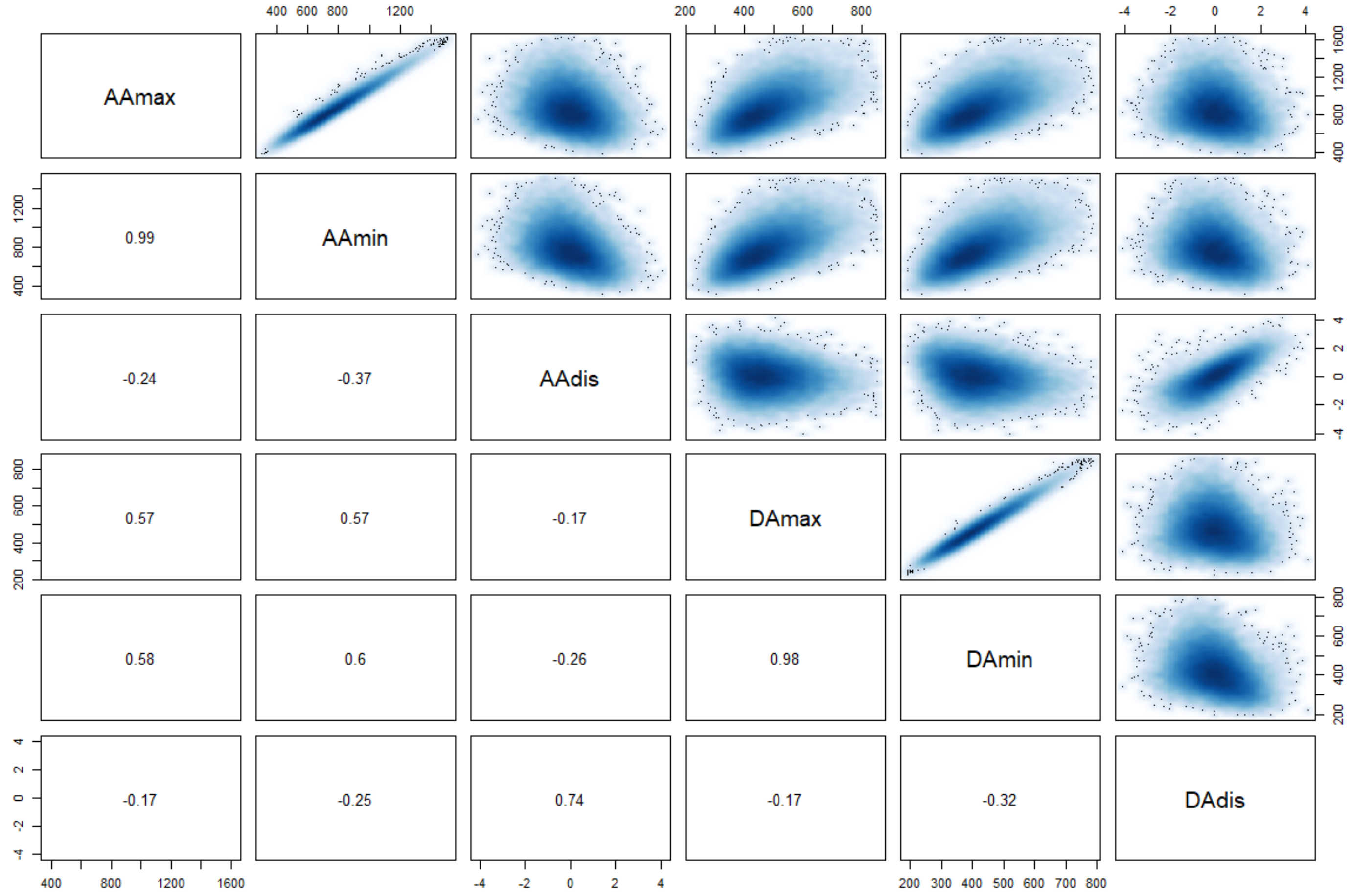

Supplementary Figure 3: Correlation between imaging phenotypes. Upper panels display smooth scatter plots, lower panels show the pair-wise Pearson's correlation coefficient (two-sided). Plots or coefficients for distensibility phenotypes are based on rank normalised data. $p<2.2x10^{-16}$ for all comparisons; for detailed results see Supplementary Table 3

Supplementary Figure 4

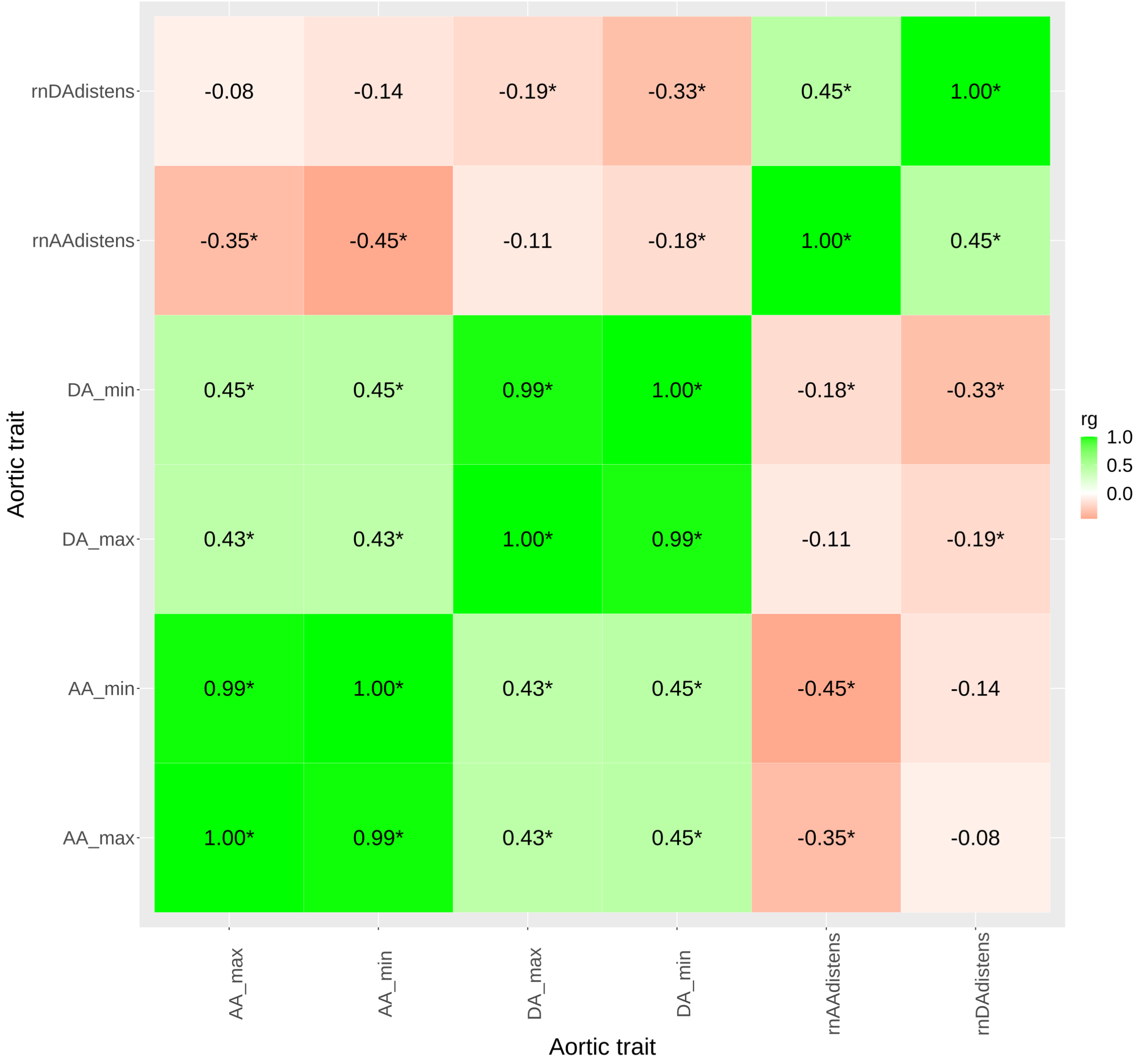

Supplementary Figure 4. Genetic correlation between traits using LD score regression (see Methods for details). Correlation coefficients are presented (r_g), with green representing positive correlation and red a negative correlation coefficient. AA_max: maximum ascending aortic area; AA_min: minimum ascending aortic area; DA_max: maximum descending aortic area; DA_min: minimum descending aortic area; rnAAdistens: rank-normalised ascending aortic distensibility; rnDAdistens: rank-normalised descending aortic distensibility. Asterisk indicates p value (uncorrected) of correlation <0.05. Exact p values and full results can be found in Supplementary Table 4

Supplementary Figure 5: Manhattan plots - Stage 1 GWAS

a

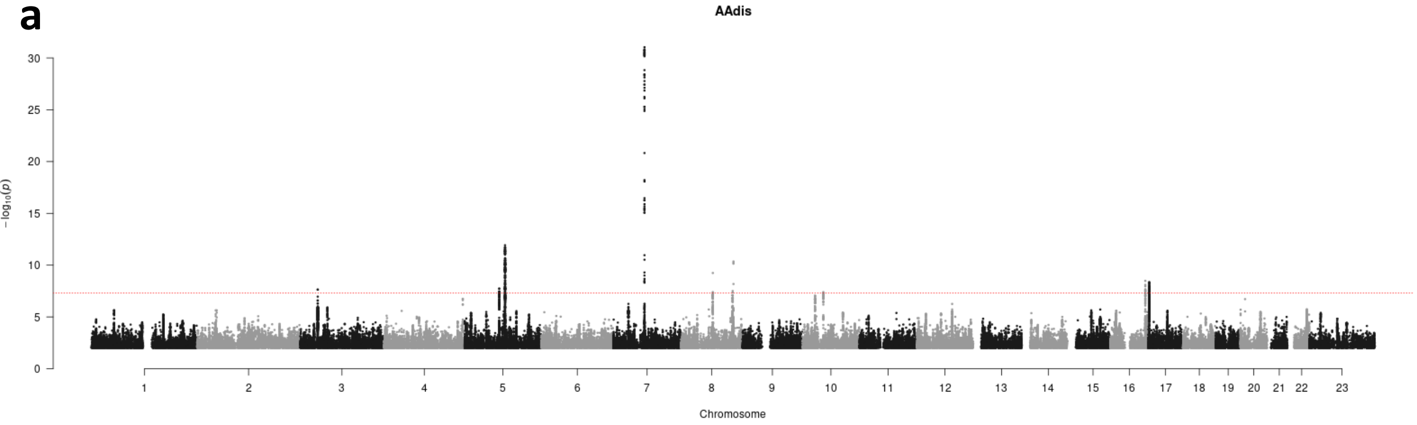

b

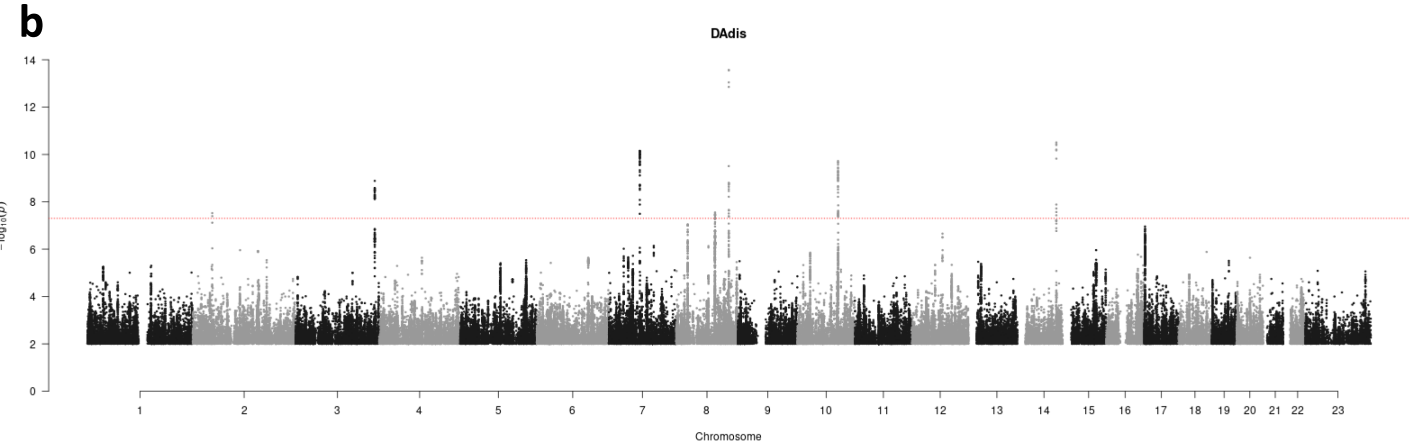

c

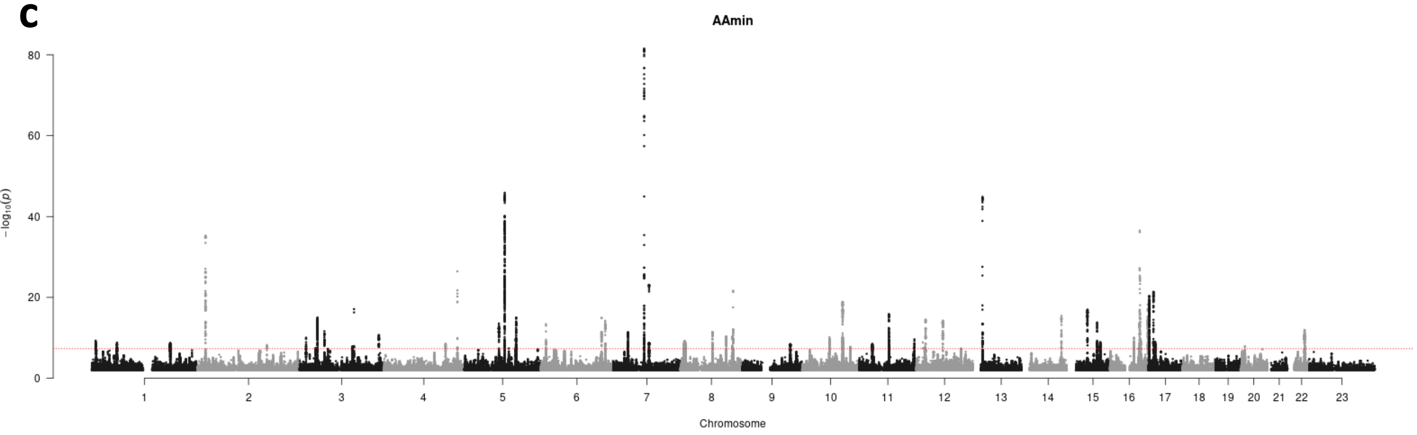

d

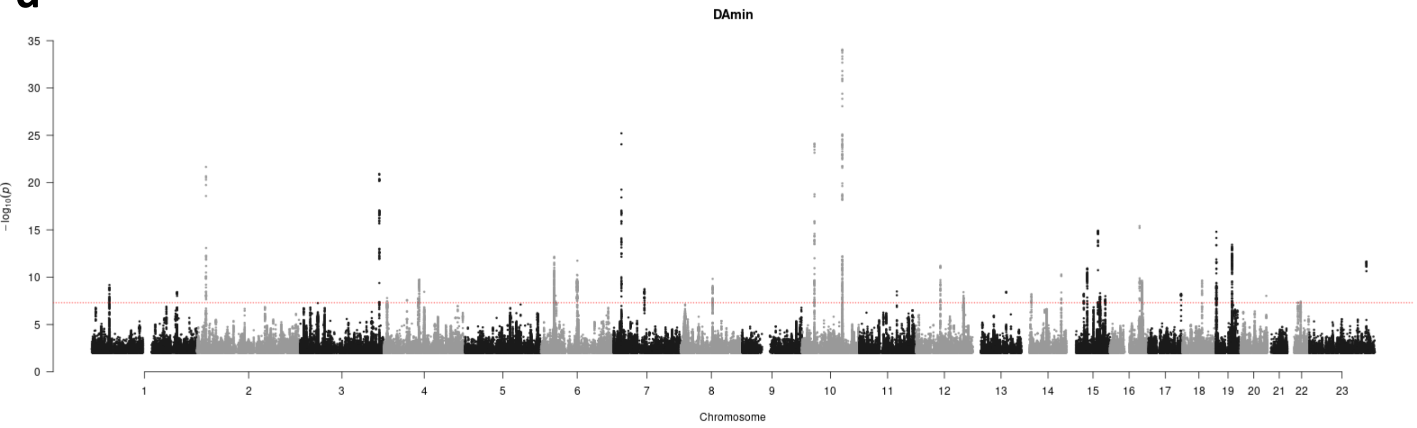

Supplementary Figure 5 cont…

e

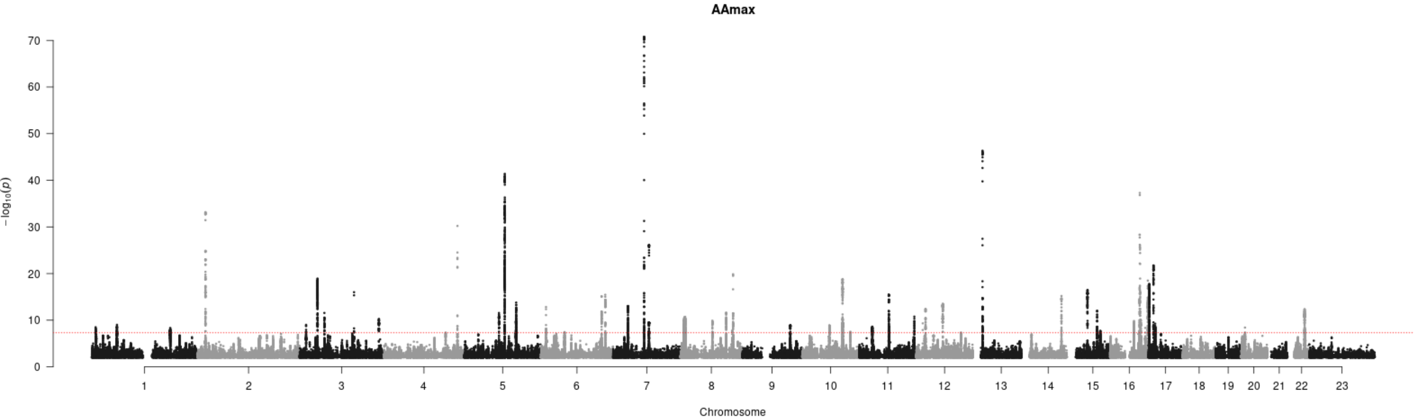

f

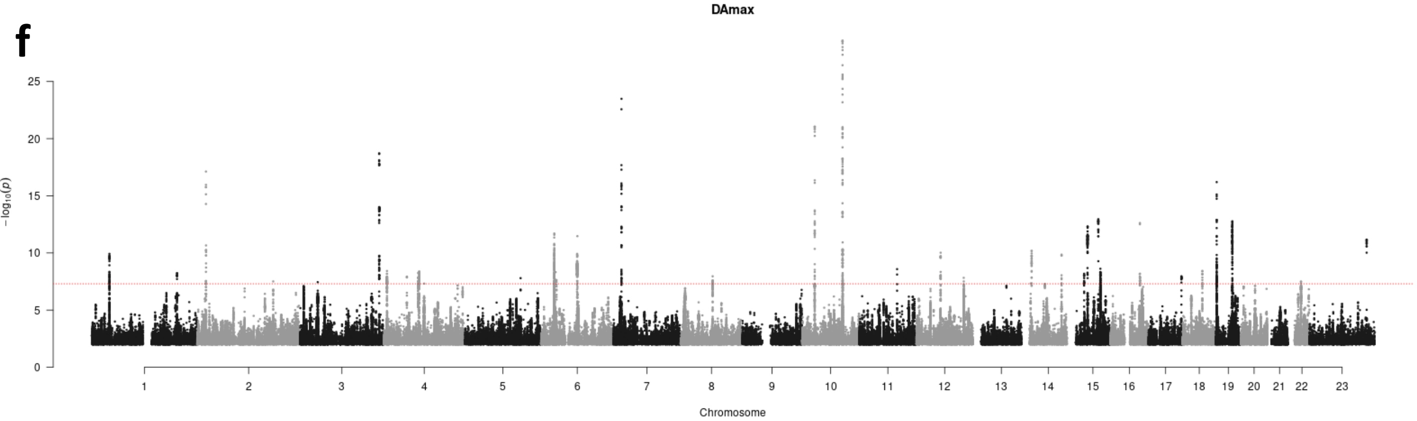

Supplementary Figure 5: Single-trait genome-wide analysis results were obtained using BOLT-LMM. Summary statistics (P values from infinitesimal mixed model associations as described in Methods) are shown as Manhattan plots with red dashed line showing the genome-wide significance threshold of $P = 5 \times 10^{-8}$. a= ascending aortic distensibility (AAdis), b=descending aortic distensibility (DAdis), c= ascending aortic minimum area (AAmin), d= descending aortic minimum area (DAmin), e= ascending aortic maximum area (AAmax), f= descending aortic maximum area (DAmax).

Supplementary Figure 6: QQ plots - Stage 1 GWAS

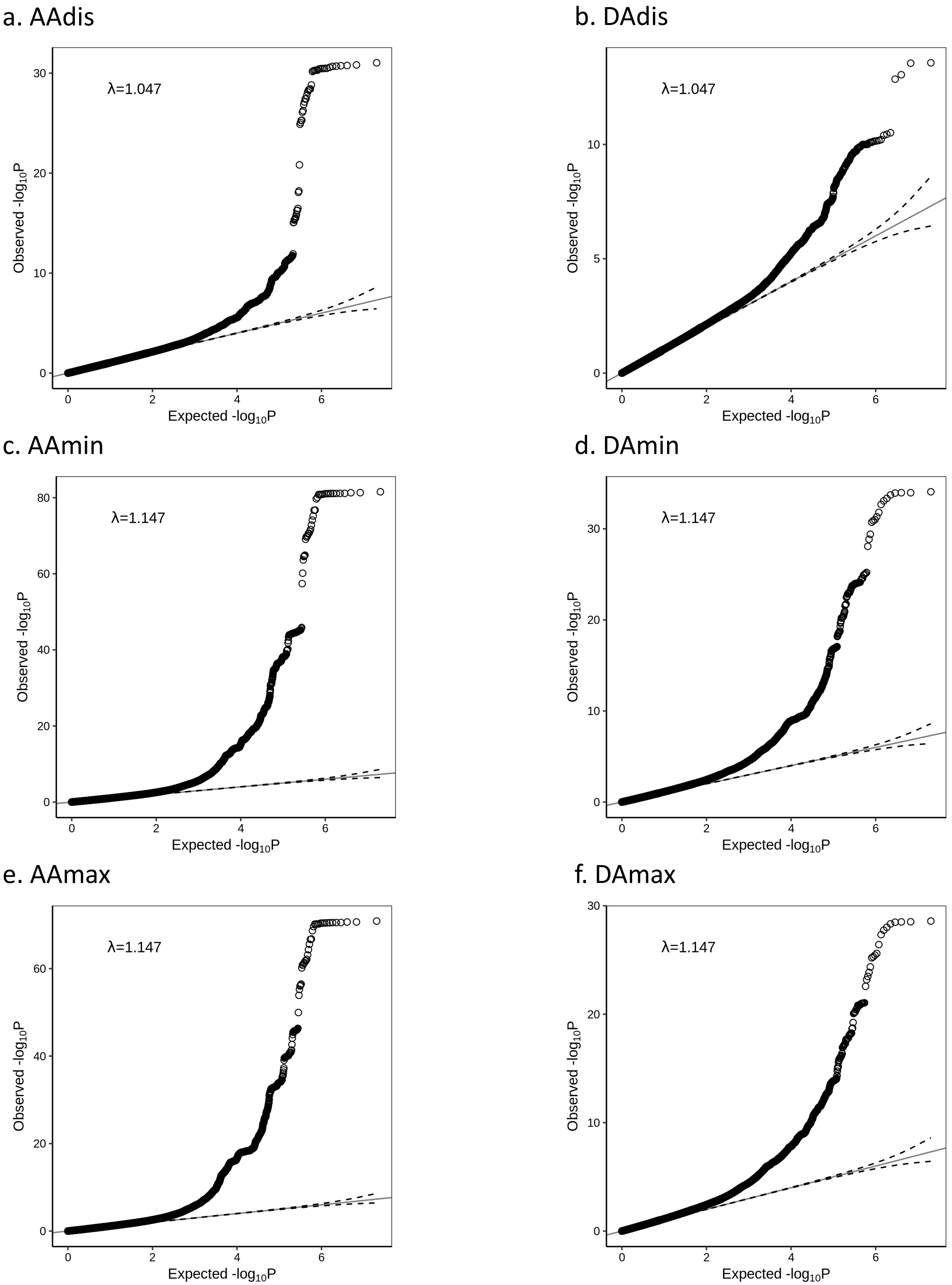

Supplementary Figure 6: QQ plots showing observed versus expected -$\log_{10}$ p values for stage 1 GWAS (from infinitesimal mixed model associations using BOLT-LMM), with genomic inflation factor in insert. a= ascending aortic distensibility (AAdis), b=descending aortic distensibility (DAdis), c= ascending aortic minimum area (AAmin), d= descending aortic minimum area (DAmin), e= ascending aortic maximum area (AAmax), f= descending aortic maximum area (DAmax).

Supplementary Figure 7: Manhattan plots - Stage 2 (MTAG) GWAS

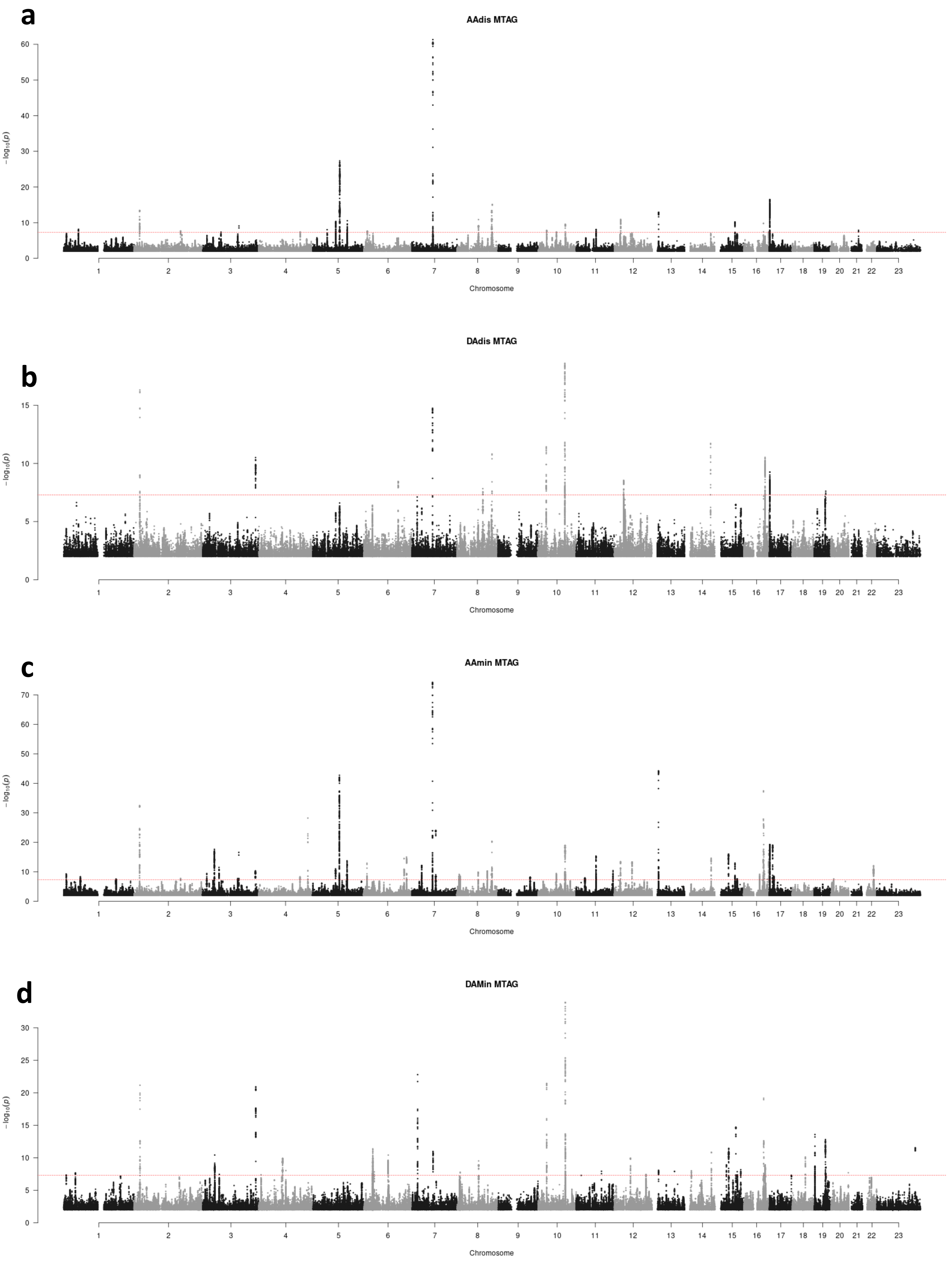

Supplementary Figure 7 cont…

e

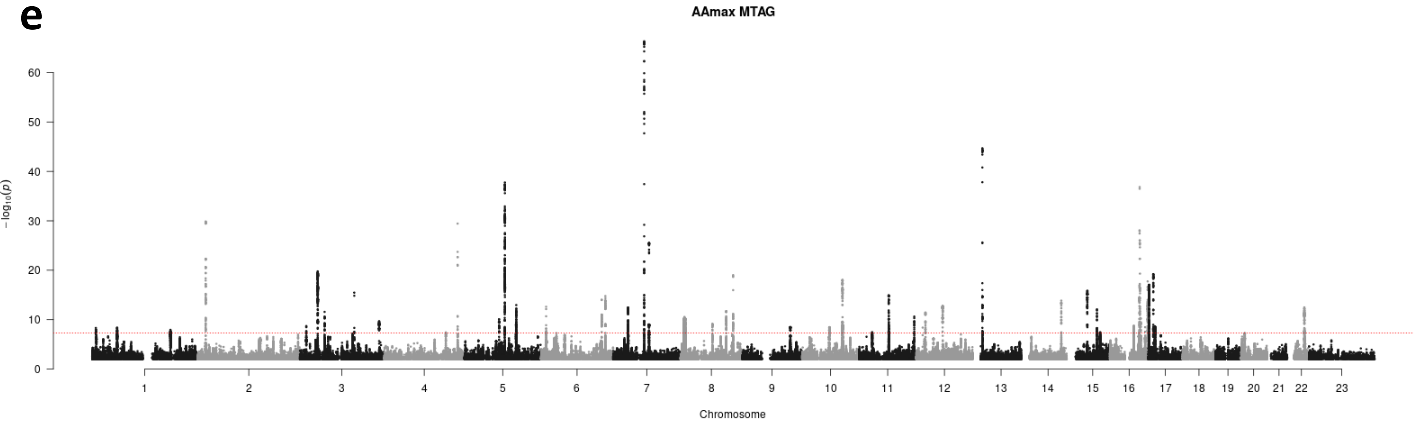

f

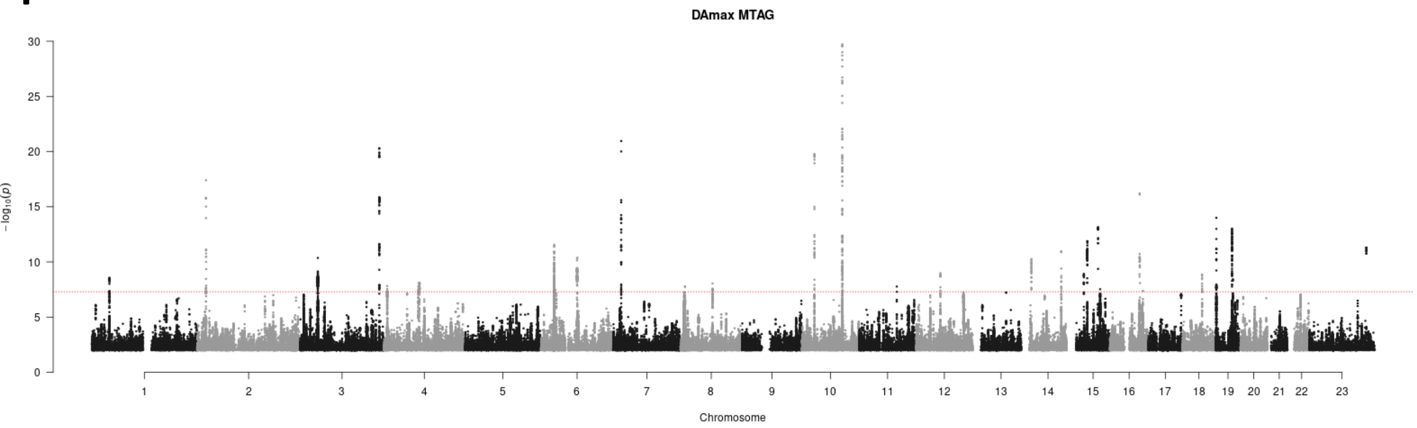

Supplementary Figure 7 Manhattan plots showing multi-trait (MTAG) GWAS results using all six aortic traits..Unadjusted p values shown are derived from MTAG analysis as described in Methods. Summary statistics from MTAG are shown as Manhattan plots with red dashed line showing the genome-wide significance threshold of $P = 5 \times 10^{-8}$. a= AAdis, b=DAdis, c=AAmin, d=DAmin, e=AAmax, f=DAmax.

Supplementary Figure 8

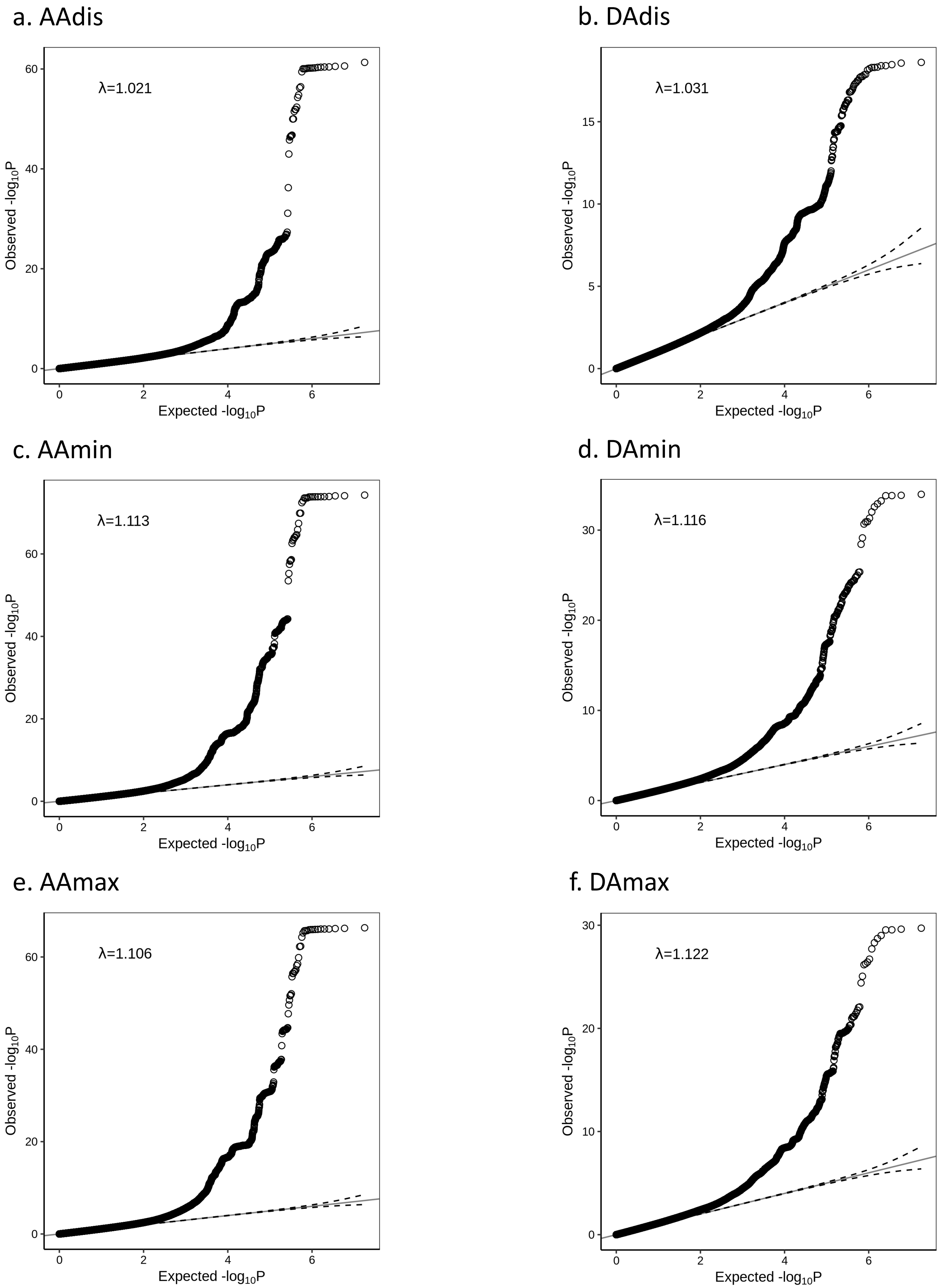

Supplementary Figure 8: QQ plots showing observed versus expected -log$_{10}$ p values (unadjusted) for stage 2 (multi-trait) GWAS analysis using MTAG, with genomic inflation factor (λ) in insert. Please see Methods for details of analysis. a= ascending aortic distensibility (AAdis), b=descending aortic distensibility (DAdis), c= ascending aortic minimum area (AAmin), d= descending aortic minimum area (DAmin), e= ascending aortic maximum area (AAmax), f= descending aortic maximum area (DAmax).

Supplementary Figure 9

a

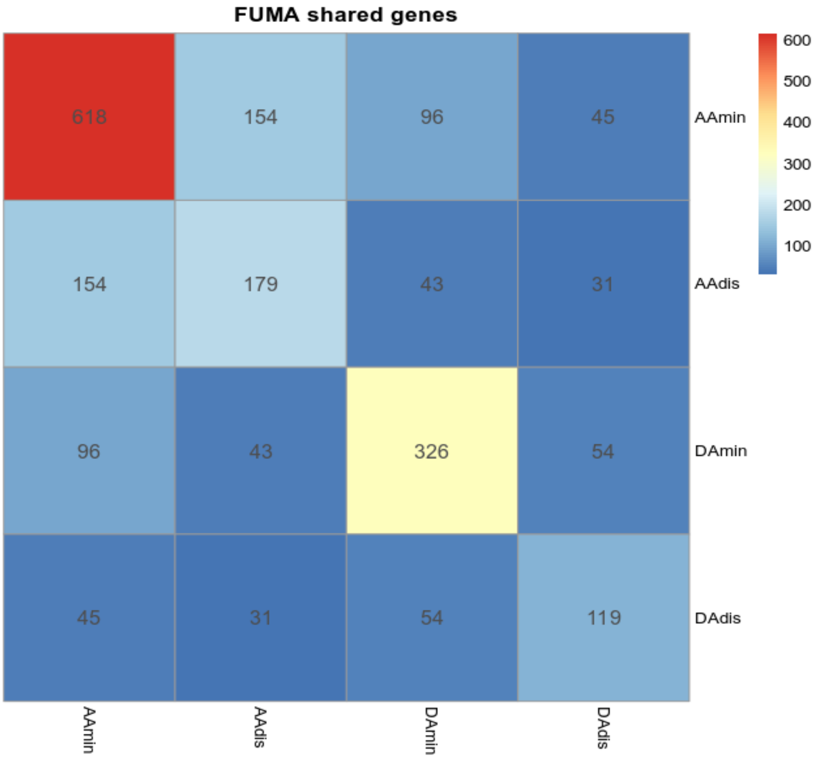

b

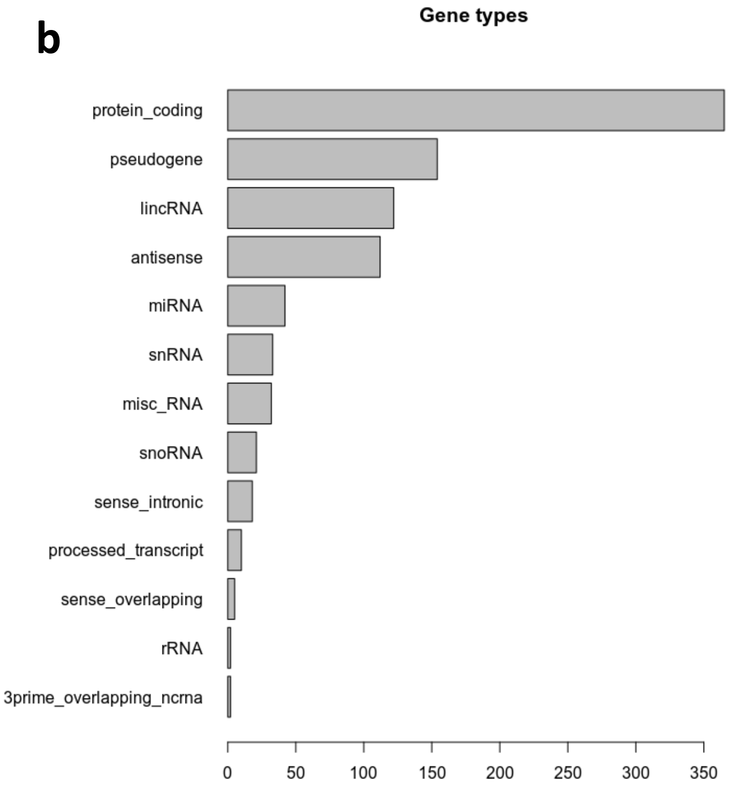

Supplementary Figure 9: FUMA results: a) Overlap of genes detected for FUMA for different traits; b) Gene types detected by FUMA

<u>Supplementary Figure 10</u>

a. AAdis (MTAG)

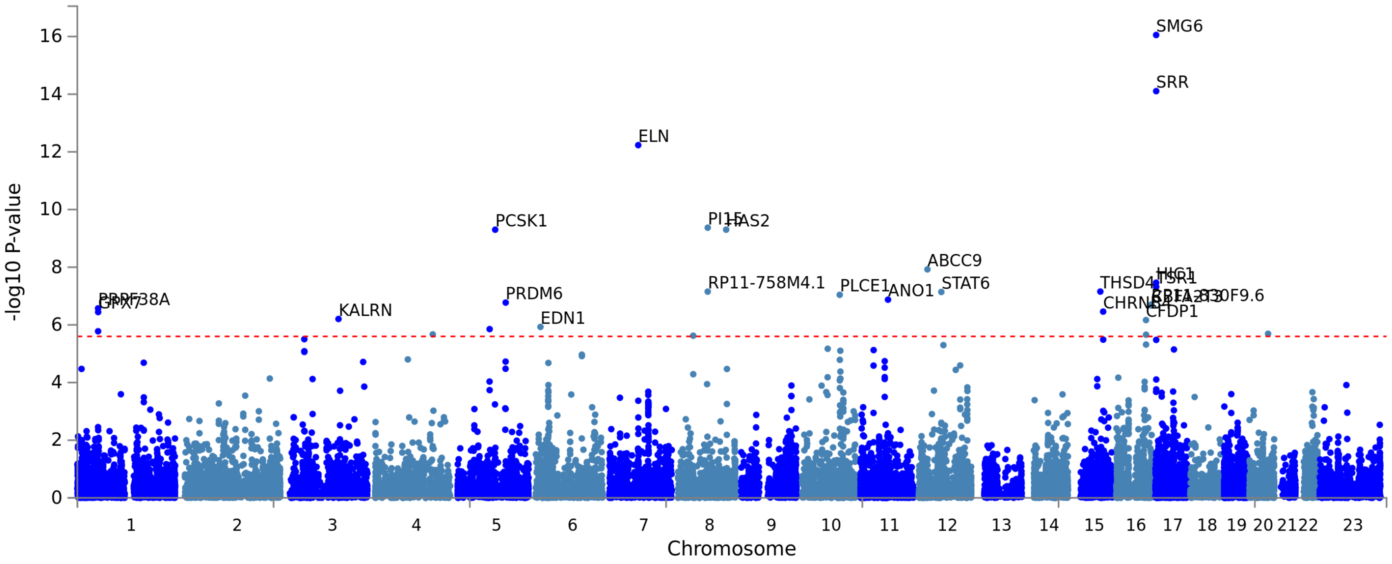

b. DAdis (MTAG)

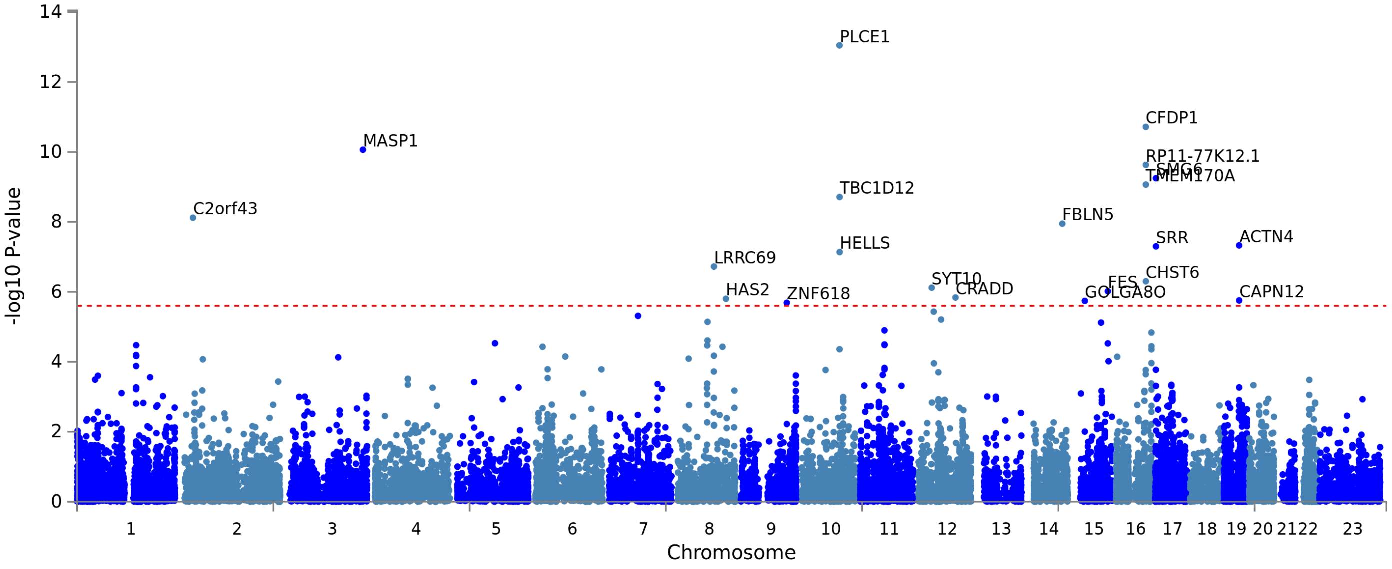

Supplementary Figure 10: Manhattan plots showing gene-based analysis results from MAGMA (implemented in FUMA). The top 21 genes associated with each trait are labelled. a) Ascending aortic distensibility (AAdis); b) Descending aortic distensibility (DAdis)

Supplementary Figure 10 cont…

c. AAmin

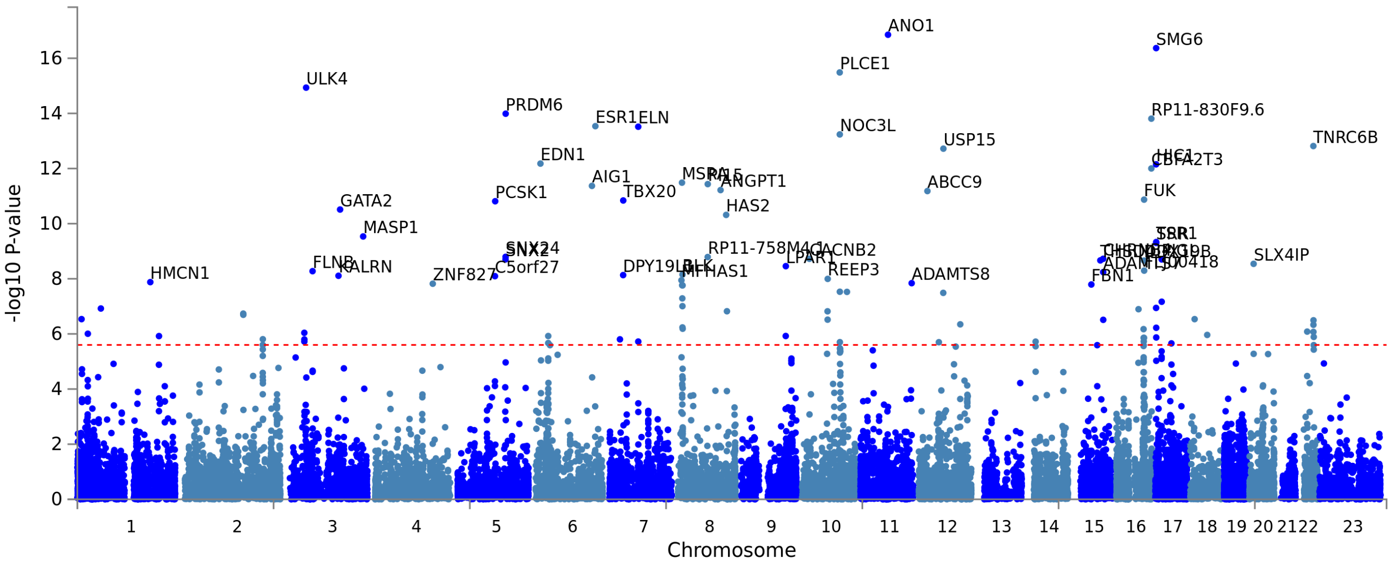

d. DAmin

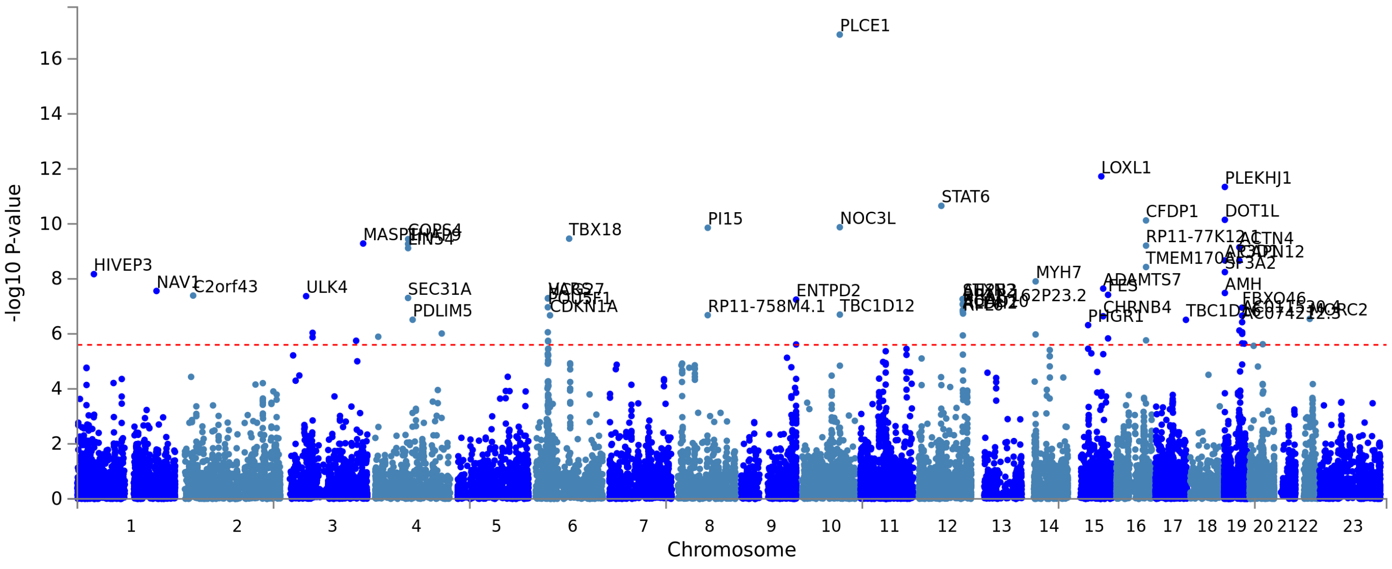

Supplementary Figure 10 cont: Manhattan plots showing gene-based analysis results from MAGMA (implemented in FUMA). The top 50 genes associated with each trait are labelled. c) Ascending aortic minimum area (AAmin); d) Descending aortic minimum area (DAmin)

Supplementary Figure 11

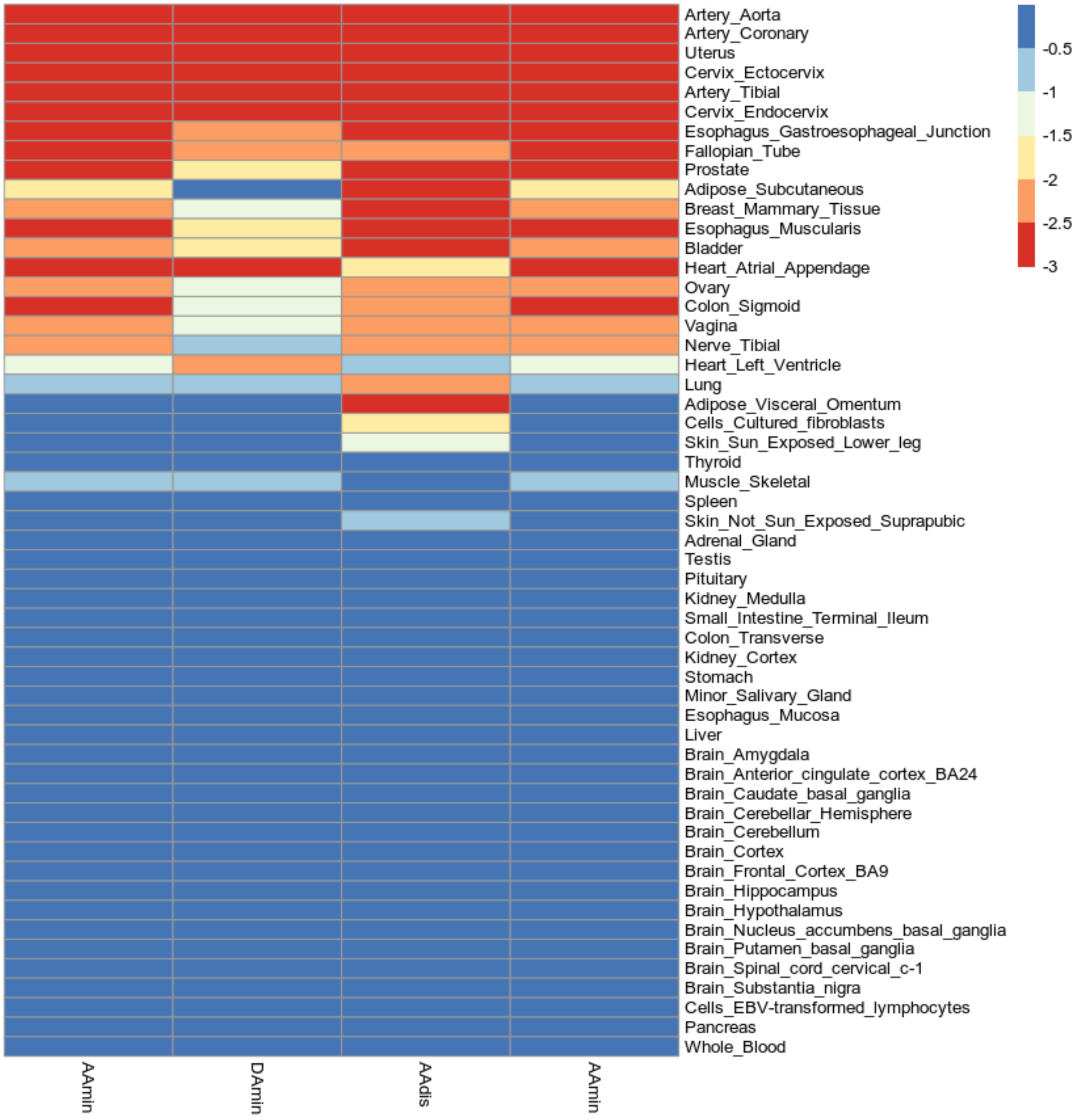

Supplementary Figure 11: Heat map showing tissue expression analysis, with the scale showing the –log10 p value of enrichment. Analysis in MAGMA, implemented in FUMA.

Supplementary Figure 12

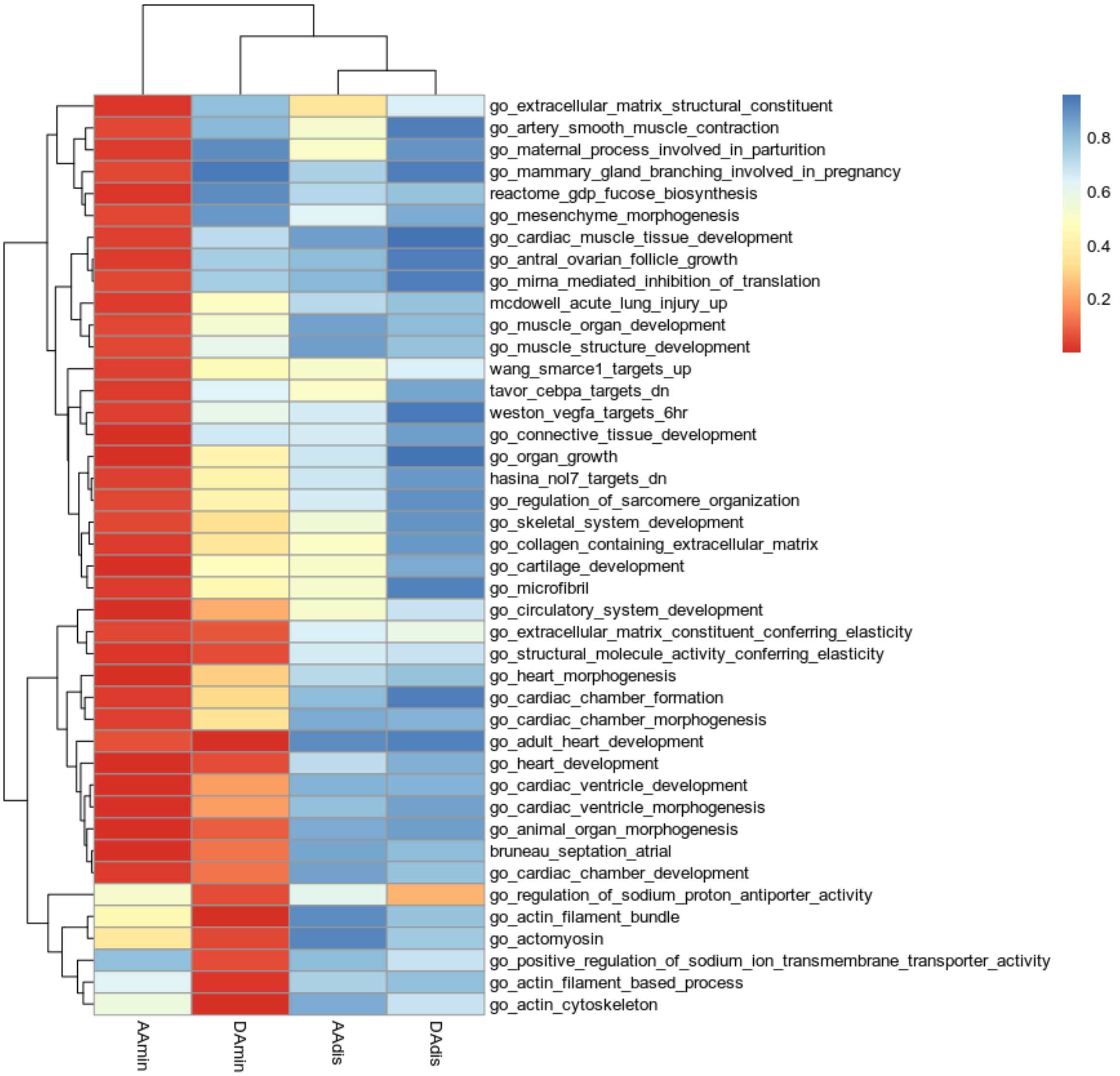

Supplementary Figure 12: MAGMA gene set enrichment Threshold FDR < 0.05 for at least one trait. Colour scale shows p value, adjusted for multiple comparisons in MAGMA.

Supplementary Figure 13

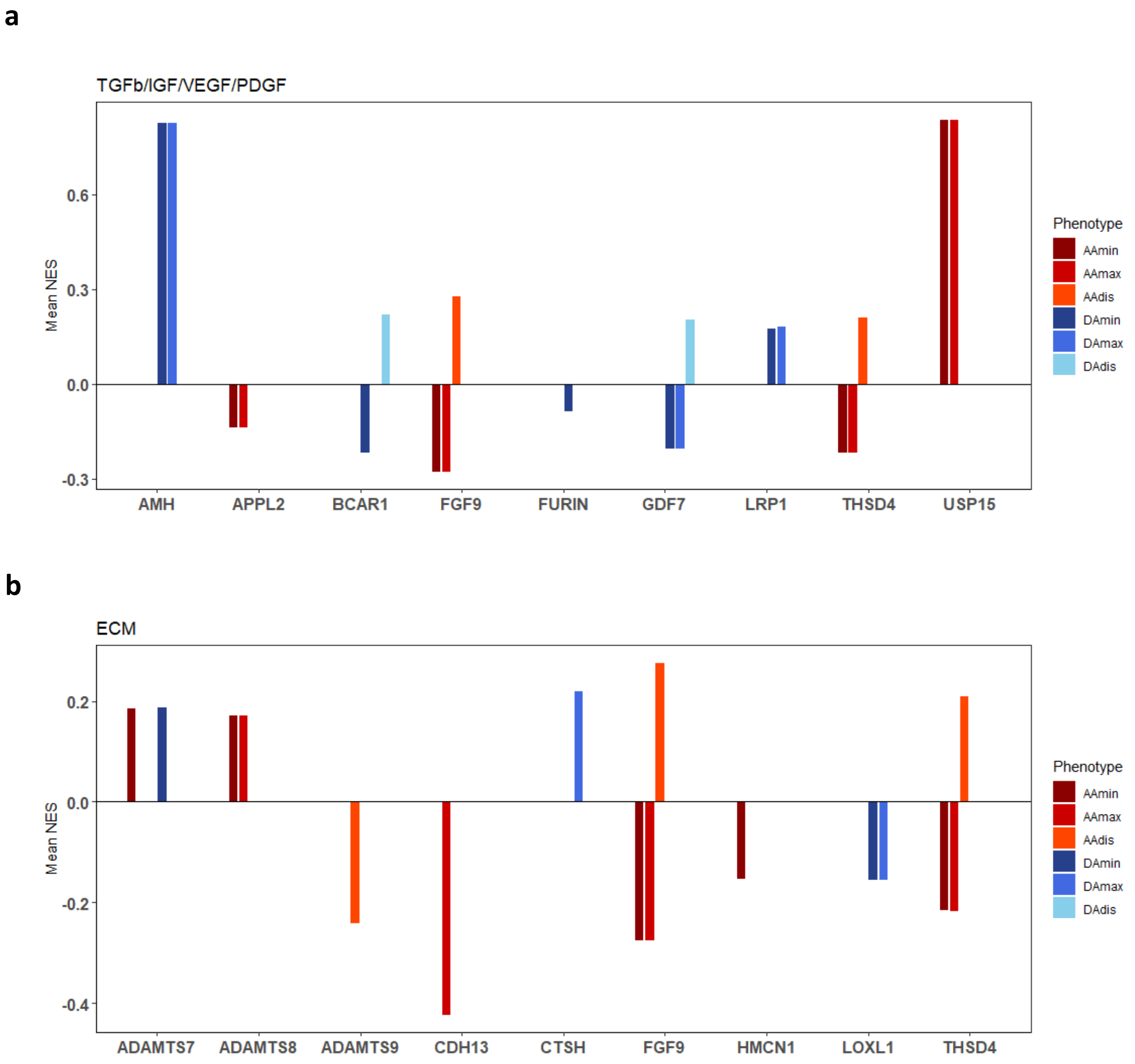

Supplementary Figure 13: Average normalised effect size (NES) of eQTLs mapped to selected candidate genes by FUMA. NES for the different imaging phenotypes is indicated by colour. **a)** NES for genes associated with TGF-b, IGF, VEGF or PDGF pathways based on the KEGG (hsa04350, hsa04910 , hsa04370, hsa04512) and GO databases (GO:0048009, GO:0048010, GO:0048008, GO:0007179). **b)** NES for genes associated with the extracellular matrix (ECM) by KEGG (hsa04512) and GO (GO:0031012) databases.

Supplementary Figure 14

a

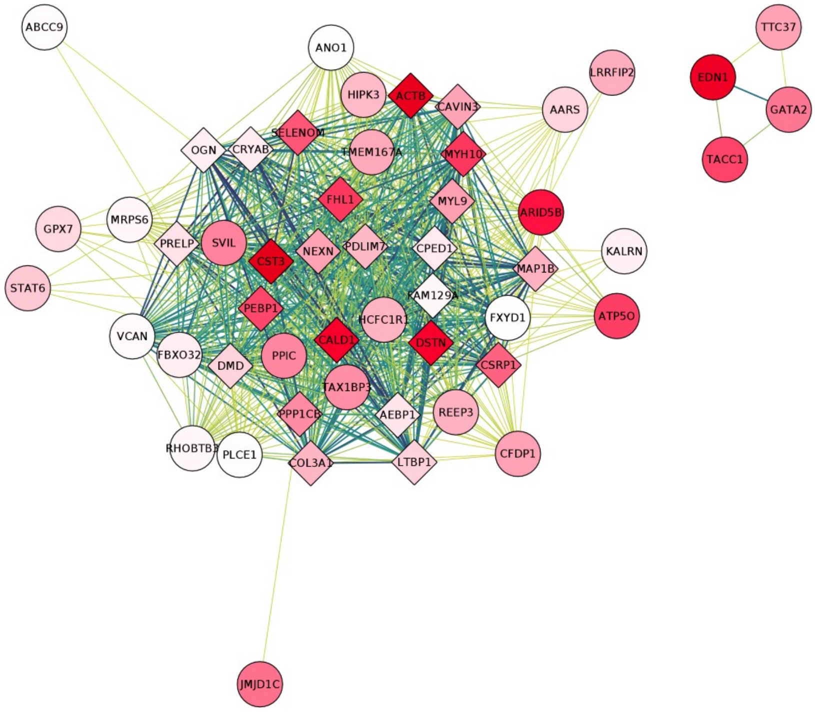

b

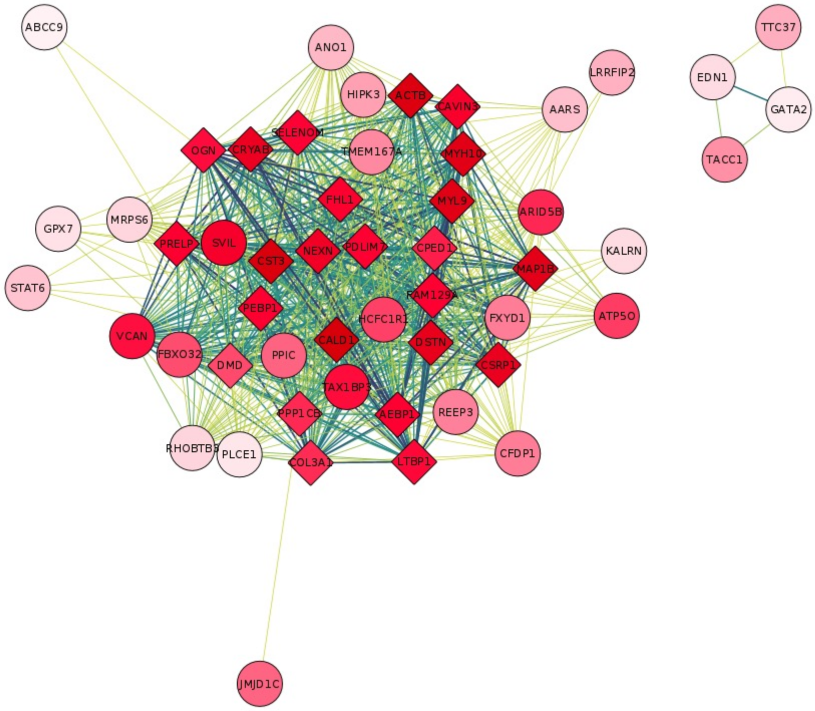

Supplementary Figure 14: Extended coexpression networks: AAdis (MTAG) Co-expression networks for aortic GWAS genes generated with primate single cell expression data for the aorta[33]. Shown are co-expression networks derived from extended models (r>0.1) in a) endothelial and b) smooth muscle cells. Round circles represent genes which were significantly associated with an aortic trait in the current GWAS. Diamonds represent other genes significantly co-expressed in the published single cell data for the cell-type indicated. The deeper the shade of red, the more highly that gene is expressed in the cell-type. The strength of co-expression is denoted by the colour of the lines joining genes with higher correlations indicated by darker lines. Hub genes are therefore found in the centres of these modules.

Supplementary Figure 15

a

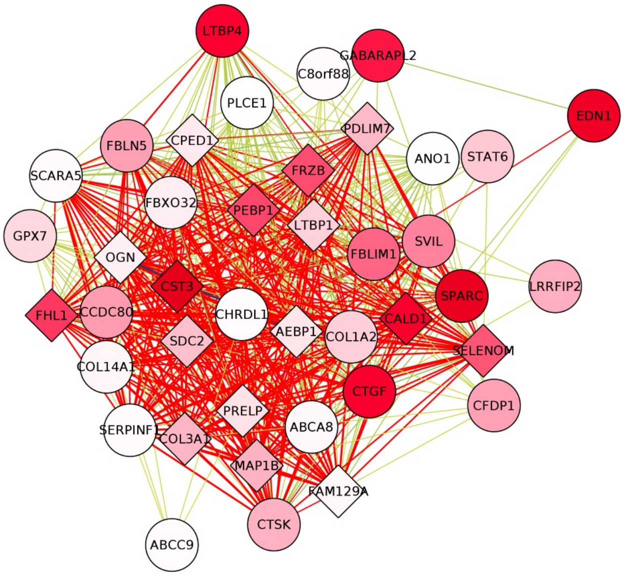

b

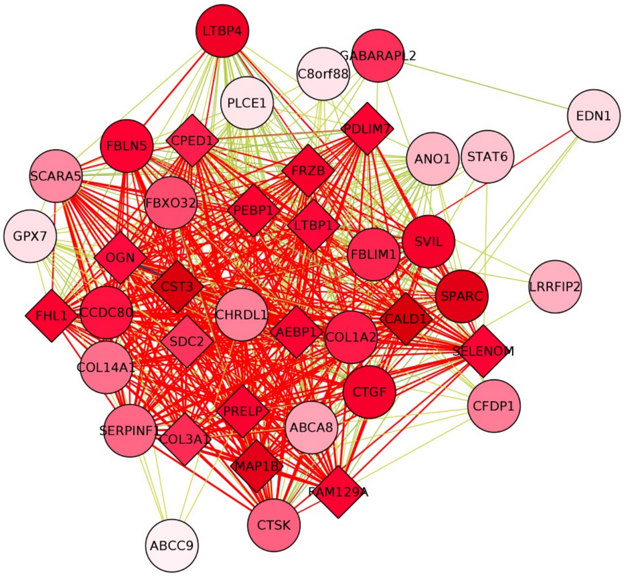

Supplementary Figure 15: Extended coexpression networks: DAdis (MTAG). Co-expression networks for aortic GWAS genes generated with primate single cell expression data for the aorta[33]. Shown are co-expression networks derived from extended models (r>0.1) in a) endothelial and b) smooth muscle cells. Round circles represent genes which were significantly associated with an aortic trait in the current GWAS. Diamonds represent other genes significantly co-expressed in the published single cell data for the cell-type indicated. The deeper the shade of red, the more highly that gene is expressed in the cell-type. The strength of co-expression is denoted by the colour of the lines joining genes with higher correlations indicated by darker lines. Hub genes are therefore found in the centres of these modules.

<u>**Supplementary Figure 16**</u>

a

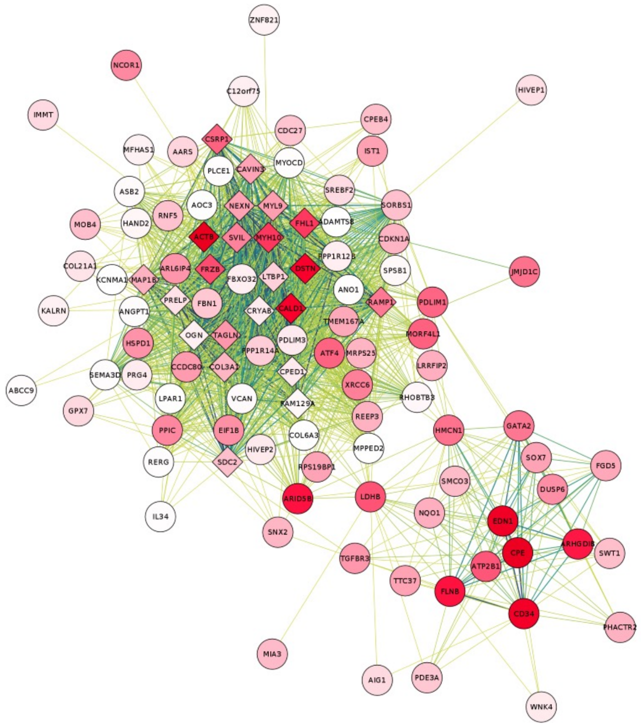

b

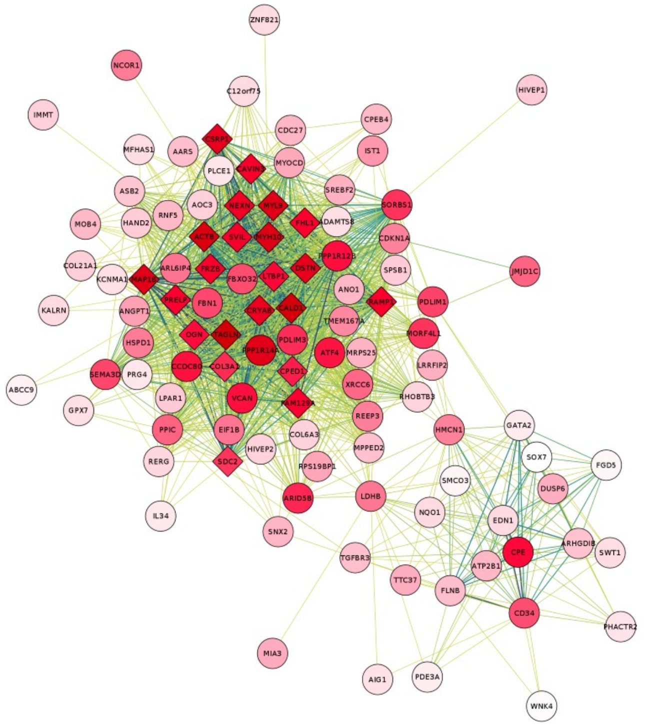

Supplementary Figure 16: Extended coexpression networks: AAmin. Co-expression networks for aortic GWAS genes generated with primate single cell expression data for the aorta[33]. Shown are co-expression networks derived from extended models (r>0.1) in a) endothelial and b) smooth muscle cells. Round circles represent genes which were significantly associated with an aortic trait in the current GWAS. Diamonds represent other genes significantly co-expressed in the published single cell data for the cell-type indicated. The deeper the shade of red, the more highly that gene is expressed in the cell-type. The strength of co-expression is denoted by the colour of the lines joining genes with higher correlations indicated by darker lines. Hub genes are therefore found in the centres of these modules.

Supplementary Figure 17

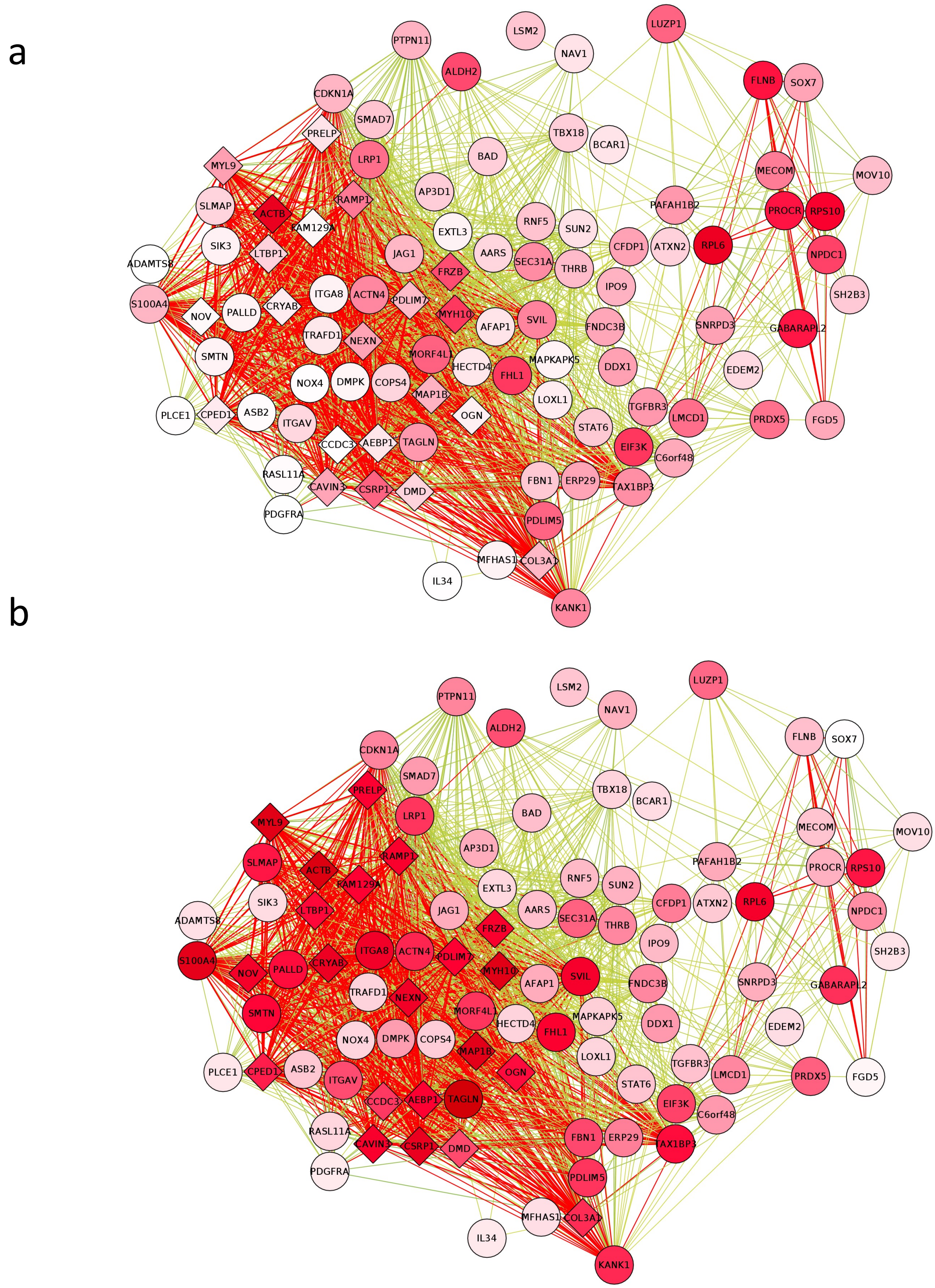

Supplementary Figure 17: Extended coexpression networks: DAmin. Co-expression networks for aortic GWAS genes generated with primate single cell expression data for the aorta[33]. Shown are co-expression networks derived from extended models (r>0.1) in a) endothelial and b) smooth muscle cells. Round circles represent genes which were significantly associated with an aortic trait in the current GWAS. Diamonds represent other genes significantly co-expressed in the published single cell data for the cell-type indicated. The deeper the shade of red, the more highly that gene is expressed in the cell-type. The strength of co-expression is denoted by the colour of the lines joining genes with higher correlations indicated by darker lines. Hub genes are therefore found in the centres of these modules.

Supplementary Figure 18

a

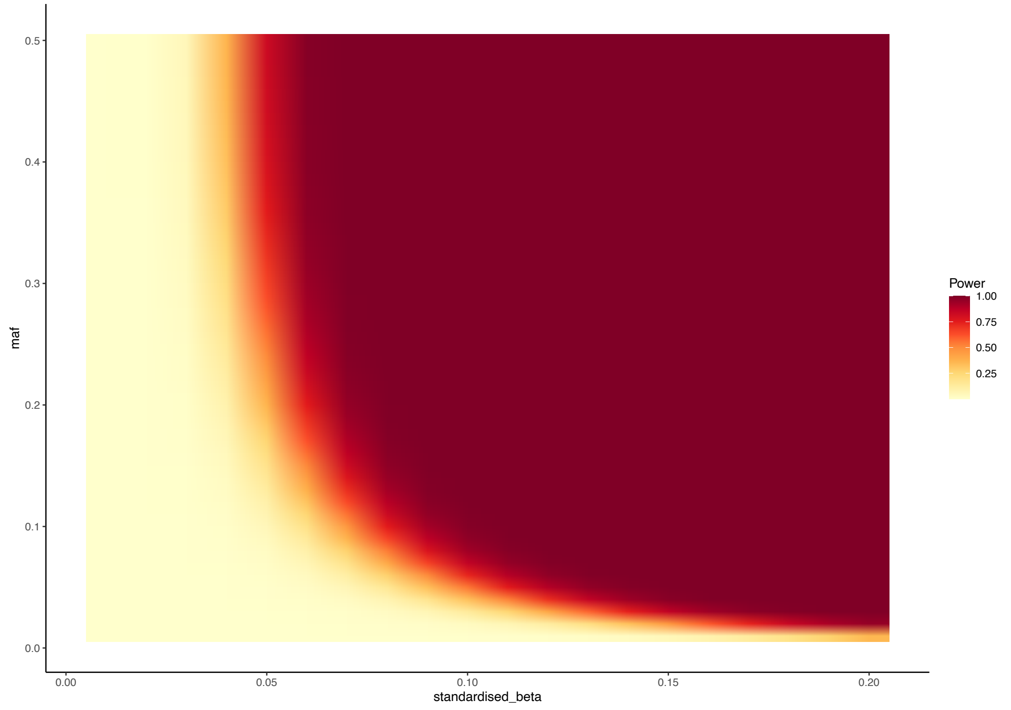

b

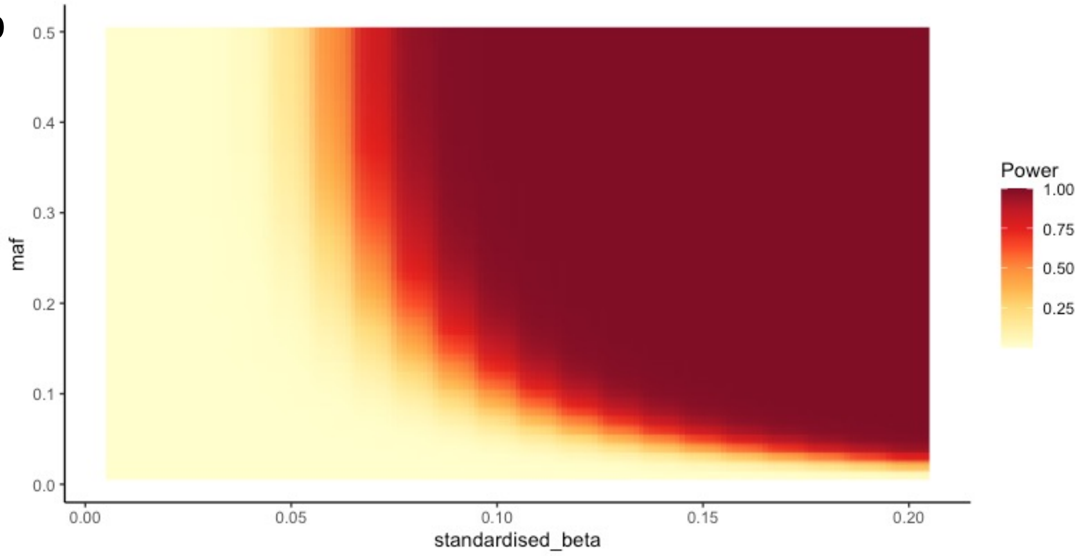

Supplementary Figure 18: Power heatmaps showing a) Power given a sample size of 32590 to detect genome-wide significant associations at a range of standardized betas and minor allele frequencies. b) Power calculations as above, given a sample size of 15817, our smallest sample size for males in sex-specific GWAS. Power calculations performed using the gwas -power functions in R as described in Visscher et al [80]

Supplementary Information: Replication cohort methods: Study of Health in Pomerania (SHIP)

Study Population

SHIP is a population-based project in West Pomerania, a region in the northeast of Germany, that consists of two independent prospectively collected cohorts (SHIP and SHIP-Trend) assessing the prevalence and incidence of common population-based diseases and their risk factors. The study design has been previously described in detail[1]. Briefly, a sample from the population aged 20 to 79 years was drawn from population registries. First, the three cities of the region (with 17,076 to 65,977 inhabitants) and the 12 towns (with 1,516 to 3,044 inhabitants) were selected, and then 17 out of 97 smaller towns (with less than 1,500 inhabitants), were drawn at random. Second, from each of the selected communities, subjects were drawn at random, proportional to the population size of each community and stratified by age and gender. Only individuals with German citizenship and main residency in the study area were included.

For SHIP, baseline examinations were carried out from 1997 until 2001, and the sample finally comprised 4,308 participants. Baseline examinations for SHIP-Trend were carried out between 2008 and 2012, finally comprising 4420 participants. Whole-body MRI was assessed in the second 5-year follow-up of SHIP (SHIP-2) and in SHIP-Trend, and subsequently included in this project.

The medical ethics committee of the University of Greifswald approved the study protocol, and oral and written informed consents were obtained from each of the study participants.

MR Imaging

MR imaging was performed on a 1.5-T MR system (Magnetom Avanto; Siemens Medical Systems, Erlangen, Germany) using integrated coil elements and phased-array surface coils. The axial 3D-T1-VIBE sequences (volume interpolated breath-hold examination) with ECG triggering and breath-hold technique were used to display the ascending and descending aorta (field of view: 450 mm x 365 mm, matrix: 256 x 256). Additionally, the following sequences were included in the reading procedure: T1-VIBE thorax (TR of 3.1 ms; TE of 1.1 ms; 8° flip angle; voxel size of 1.8 × 1.8 × 3.0 mm; scan time 21s) and abdomen (2 image stacks, TR of 7.5 ms; TE of 2.4 ms; 10° flip angle; voxel size of 2.4 × 1.6 × 4.0 mm; scan time 38s).

The post-processing and measurements of the vessel diameters were carried out with the image viewing and editing software OsiriX (version 3.6.1, 64-bit; http://www.osirix-viewer.com). For this purpose, eight measurement points were defined. Measurement was taken from vessel outer wall to outer wall (outer diameter). For oval vessels, the smallest vessel diameter was selected.

Reading was performed by two trained and certified observers. The quality of the diameter reading was assigned using four categories: very good, good, difficult to measure, and not measurable. Results of the latter category were excluded from the analyses.

Genotyping

Nonfasting blood samples were drawn from the cubital vein in the supine position. The samples were taken between 07:00 AM and 04:00 PM, and serum aliquots were prepared for immediate analysis and for storage at -80 °C in the Integrated Research Biobank (Liconic, Liechtenstein).

The SHIP samples were genotyped using the Affymetrix Genome-Wide Human SNP Array 6.0. Hybridisation of genomic DNA was done in accordance with the manufacturer's standard recommendations. Genetic data were stored using the database Caché (InterSystems). Genotypes were determined using the Birdseed2 clustering algorithm. For quality control purposes, several control samples where added. On the chip level, only subjects with a genotyping rate on QC probesets (QC callrate) of at least 86% were included. Finally, all arrays had a sample callrate > 92%. Duplicate samples (based on estimated IBD), and mismatches between reported and genotyped sex were removed, leaving 4070 arrays for subsequent analyses. SNPs with a Hardy-Weinberg-Equilibrium p-value <0.0001, a call rate <0.95, and monomorphic SNPs were removed before imputation, as well as SNPs having position mapping problem from genome build b36 to b37, or duplicate IDs.

A subset of the SHIP-Trend samples was genotyped using the Illumina Human Omni 2.5 array. Hybridisation of genomic DNA was done in accordance with the manufacturer's standard recommendations at the Helmholtz Zentrum München. Genotypes were determined using the GenomeStudio Genotyping Module v1.0 (GenCall algorithm) or the GenomeStudio 2.0 Genotyping Module (GenCall algorithm). Arrays with a genotyping call rate <94%, duplicates (based on estimated IBD), and mismatches between reported and genotyped sex were removed, leaving 986 arrays for subsequent analyses. SNPs with a Hardy-Weinberg-Equilibrium p-value <0.0001, a call rate <0.95, or monomorphic SNPs were removed before imputation, as well as SNPs having position mapping problem from genome build b36 to b37, duplicate IDs.

The remaining SHIP-Trend samples was genotyped using the Illumina GSA chip. Hybridisation of genomic DNA was done in accordance with the manufacturer's standard recommendations at the LIFE & BRAIN GmbH, Bonn, Germany. Genotypes were determined using the GenomeStudio 2.0 Genotyping Module (GenCall algorithm). Prior to subsequent quality control, SNPs with a MAC ≤10 were excluded. Arrays with a genotyping call rate <94%, duplicates (based on estimated IBD), mismatches between reported and genotyped sex, genetic PCA outliers (>8 SD of the mean in one of the first 10 PCs in 5 iterations), and arrays with extreme heterozygosity (>4 SD of the mean) were removed, leaving 3,133 arrays for subsequent analyses. SNPs with a Hardy-Weinberg-Equilibrium p-value <0.0001, a call rate <0.95, and a MAF <1% or a minor allele count <10 were removed before imputation.

Imputation of the genotypes was performed using the HRCv1.1 reference panel and the Eagle and minimac3 software implemented in the Michigan Imputation Server for pre-phasing and imputation, respectively. Only genotyped SNPs with consistent reference site alleles were included in the imputation process.

Statistical Analyses

In total, MRI and genetic data was available from 986 SHIP and 1801 SHIP-Trend participants for subsequent analyses. Genome-wide linear regression analyses were performed in each cohort separately using EPACTS-3.2.9 (https://github.com/statgen/EPACTS) adjusted for sex, age, mean arterial pressure, body height, body weight, array type (SHIP-Trend only), and the first two genetic principal components. The results of both cohorts were subsequently meta-analyzed using an inverse-variance weighted method implemented in METAL[2].

Acknowledgements

SHIP is part of the Community Medicine Research net of the University of Greifswald, Germany, which is funded by the Federal Ministry of Education and Research (grants no. 01ZZ9603, 01ZZ0103, and 01ZZ0403), the Ministry of Cultural Affairs as well as the Social

Ministry of the Federal State of Mecklenburg-West Pomerania, and the network ‘Greifswald Approach to Individualized Medicine (GANI_MED)’ funded by the Federal Ministry of Education and Research (grant 03IS2061A). Whole-body MR imaging was supported by a joint grant from Siemens Healthineers, Erlangen, Germany and the Federal State of Mecklenburg West Pomerania. The University of Greifswald is a member of the Caché Campus program of the InterSystems GmbH.

1. Volzke H, Alte D, Schmidt CO, Radke D, Lorbeer R, Friedrich N, et al. Cohort profile: the study of health in Pomerania. Int J Epidemiol. 2011;40(2):294-307.
2. Willer CJ, Li Y, Abecasis GR. METAL: fast and efficient meta-analysis of genomewide association scans. Bioinformatics. 2010;26(17):2190-1.
