# Supplementary material for: Genome-wide associations of aortic distensibility suggest causality for aortic aneurysms and brain white matter hyperintensities
Source: Nat Commun. 2022 Aug 3;13:4505. doi: 10.1038/s41467-022-32219-x (PMC9349177; doi:10.1038/s41467-022-32219-x)
Supplement: Supplementary file 3 — Description of Additional Supplementary Files [file 41467_2022_32219_MOESM3_ESM.pdf]

Description of Additional Supplementary Files

File Name: Supplementary Data 1 a&b
Description: Demographics and biometrics of study cohort

File Name: Supplementary Data 1c
Description: Correlations of aortic traits with other biometrics in our cohort

File Name: Supplementary Data 2
Description: Heritability of aortic traits (LDSC)

File Name: Supplementary Data 3
Description: Phenotype correlations

File Name: Supplementary Data 4
Description: LDSC Genotype correlations

File Name: Supplementary Data 5 (a-f)
Description: Significant stage 1 GWAS associations

File Name: Supplementary Data 6 (a-f)
Description: Significant stage 2 (MTAG) GWAS associations

File Name: Supplementary Data 7
Description: Full list of 102 loci

File Name: Supplementary Data 7a-d
Description: Replication of lead aortic area SNPs in SHIP study

File Name: Supplementary Data 8
Description: LD between lead SNPs for distensibility

File Name: Supplementary Data 9
Description: eQTL and sQTL annotations (distensibility loci)

File Name: Supplementary Data 10 (a-d)
Description: Functional annotations

File Name: Supplementary Data 11
Description: Comparison of results with Tcheandjieu, Pirruccello and Benjamins – locus overlap

File Name: Supplementary Data 12
Description: Sex-specific GWAS comparison

File Name: Supplementary Data 13
Description: FUMA gene-based association results

File Name: Supplementary Data 14

Description: MAGMA gene-based association results

File Name: Supplementary Data 15a
Description: MAGMA GO associations

File Name: Supplementary Data 15b
Description: DEPICT GO associations

File Name: Supplementary Data 16
Description: Pathways analysis of co-expression data

File Name: Supplementary Data 17
Description: PheWAS full results

File Name: Supplementary Data 18
Description: MR-PheWAS full results

File Name: Supplementary Data 19 (a-c)
Description: LDSC and bidirectional MR results aorta-BP

File Name: Supplementary Data 20
Description: MR-ConMix results

File Name: Supplementary Data 21
Description: LDSC analysis of aortic traits with cerebral phenotypes

File Name: Supplementary Data 22
Description: Regional correlations between aortic and cerebral traits

File Name: Supplementary Data 23
Description: Two-sample MR: aortic with cerebral traits

File Name: Supplementary Data 24
Description: Multivariable MR: BP and aortic with WMH

File Name: Supplementary Data 25
Description: Replication of MVMR signal in the Rhineland study
