# Supplementary material for: Genome-wide associations of aortic distensibility suggest causality for aortic aneurysms and brain white matter hyperintensities
Source: Nat Commun. 2022 Aug 3;13:4505. doi: 10.1038/s41467-022-32219-x (PMC9349177; doi:10.1038/s41467-022-32219-x)
Supplement: Supplementary file 5 — Reporting Summary [file 41467_2022_32219_MOESM5_ESM.pdf]

nature portfolio

Corresponding author(s): Paul M Matthews, Abbas Dehghan

Last updated by author(s): Jul 4, 2022

Reporting Summary

Nature Portfolio wishes to improve the reproducibility of the work that we publish. This form provides structure for consistency and transparency in reporting. For further information on Nature Portfolio policies, see our Editorial Policies and the Editorial Policy Checklist.

Statistics

For all statistical analyses, confirm that the following items are present in the figure legend, table legend, main text, or Methods section.

n/a	Confirmed	
☐	☒	The exact sample size (*n*) for each experimental group/condition, given as a discrete number and unit of measurement
☐	☒	A statement on whether measurements were taken from distinct samples or whether the same sample was measured repeatedly
☐	☒	The statistical test(s) used AND whether they are one- or two-sided<br>*Only common tests should be described solely by name; describe more complex techniques in the Methods section.*
☐	☒	A description of all covariates tested
☐	☒	A description of any assumptions or corrections, such as tests of normality and adjustment for multiple comparisons
☐	☒	A full description of the statistical parameters including central tendency (e.g. means) or other basic estimates (e.g. regression coefficient) AND variation (e.g. standard deviation) or associated estimates of uncertainty (e.g. confidence intervals)
☐	☒	For null hypothesis testing, the test statistic (e.g. *F*, *t*, *r*) with confidence intervals, effect sizes, degrees of freedom and *P* value noted<br>*Give P values as exact values whenever suitable.*
☒	☐	For Bayesian analysis, information on the choice of priors and Markov chain Monte Carlo settings
☒	☐	For hierarchical and complex designs, identification of the appropriate level for tests and full reporting of outcomes
☐	☒	Estimates of effect sizes (e.g. Cohen's *d*, Pearson's *r*), indicating how they were calculated

Our web collection on statistics for biologists contains articles on many of the points above.

Software and code

Policy information about availability of computer code

Data collection	No specific code used
Data analysis	BOLT-LMM v2.3.4, LDSC vl.01, MTAG vl.0.8, FUMA vl.3.6, bedtools v2.29.2, R v.3.6.0 and v 3.6.3, R packages gwas-power v1.0, MendelianRandomisation v0.6.0, MR-PRESSO v1, RadialMR v 1.0, PheWAS vl.2, MVMR v0.3 Bioconductor v3.1 4 packages scater v 1.14.6, scran v 1.14.6, clusterProfiler v3.14.4, DEPICT vl beta rel 194, PLINK v2.0, EPACTS-3.2.9, METAL v2011-03-25, GenomeStudio 2.0 Genotyping Module (GenCall algorithm), Michigan Imputation Server v 1.2.3, Python v3.8.10, stasmodels v0.12.2

For manuscripts utilizing custom algorithms or software that are central to the research but not yet described in published literature, software must be made available to editors and reviewers. We strongly encourage code deposition in a community repository (e.g. GitHub). See the Nature Portfolio guidelines for submitting code & software for further information.

Data

Policy information about availability of data

All manuscripts must include a data availability statement. This statement should provide the following information, where applicable:

- Accession codes, unique identifiers, or web links for publicly available datasets
- A description of any restrictions on data availability
- For clinical datasets or third party data, please ensure that the statement adheres to our policy

The results of stage 1 GWAS and MTAG generated in this study have been deposited in the Imperial College data repository at https://doi.org/10.14469/hpc/10653

and are freely available for download. Raw data from the UKBB participants can be requested from the UKBB Access Management System (https://bbams.ndph.ox.ac.uk). Other publicly available data used for annotation and analysis are available as follows: eQTL data used in this study from aortic tissue are available at the GTEx portal (v8) (https://www.gtexportal.org/home/tissue/Artery_Aorta). Hi-C aorta data used for 3D chromatin interaction mapping was based on HiC aorta data (accession number GSE87112 [https://www.ncbi.nlm.nih.gov/geo/query/acc.cgi?acc=GSE87112]). Coexpression data used in this study are available from Gene Expression Omnibus (accession number GSE117715 [https://www.ncbi.nlm.nih.gov/geo/query/acc.cgi?acc=GSE117715]).

Human research participants

Policy information about studies involving human research participants and Sex and Gender in Research.

Reporting on sex and gender

Use the terms sex (biological attribute) and gender (shaped by social and cultural circumstances) carefully in order to avoid confusing both terms. Indicate if findings apply to only one sex or gender; describe whether sex and gender were considered in study design whether sex and/or gender was determined based on self-reporting or assigned and methods used. Provide in the source data disaggregated sex and gender data where this information has been collected, and consent has been obtained for sharing of individual-level data; provide overall numbers in this Reporting Summary. Please state if this information has not been collected. Report sex- and gender-based analyses where performed, justify reasons for lack of sex- and gender-based analysis.

Population characteristics

Describe the covariate-relevant population characteristics of the human research participants (e.g. age, genotypic information, past and current diagnosis and treatment categories). If you filled out the behavioural & social sciences study design questions and have nothing to add here, write "See above."

Recruitment

Describe how participants were recruited. Outline any potential self-selection bias or other biases that may be present and how these are likely to impact results.

Ethics oversight

Identify the organization(s) that approved the study protocol.

Note that full information on the approval of the study protocol must also be provided in the manuscript.

Field-specific reporting

Please select the one below that is the best fit for your research. If you are not sure, read the appropriate sections before making your selection.

☒ Life sciences ☐ Behavioural & social sciences ☐ Ecological, evolutionary & environmental sciences

For a reference copy of the document with all sections, see nature.com/documents/nr-reporting-summary-flat.pdf

Life sciences study design

All studies must disclose on these points even when the disclosure is negative.

Sample size

For the main GWAS analyses, we used the largest sample size available in UK Biobank for Caucasian individuals with aortic imaging and genetic data. For aortic area traits, the sample size was 32590 and for distensibility, 29895. For details of power calculations, please see Methods. We used MTAG analysis to boost power. For all additional analyses, the sample size was the largest available dataset. For replication in the SHIP cohort, sample size was 2787.For associations with white matter hyperintensities, sample size was 50,970 in the combined CHARGE and CADISP cohorts and was 3317 in the Rhineland study - these are the largest datasets available to us at the present time which have the appropriate phenotype data. No data was newly collected for this study.

Data exclusions

Exclusion criteria for imaging were defined by UK Biobank, and included a range of relative contraindications to magnetic resonance imaging scanning as well as childhood onset disease and pregnancy.
For the main GWAS analysis, individuals were excluded based on following criteria: image QC fail, ICD10 or self-reported aortic diagnosis, grade IV or greater hypertension at imaging visit, extreme BMI (<16 or >40), outlying aortic phenotype (>4SDs from mean), Missing genotype data or genotype QC fail, heterozygosity / missingness (data from UK Biobank), sex mismatch, missing covariate data, non-Caucasian ethnicity (self-reported). We excluded SNPs failing UK Biobank protocols (filtered per batch by Hardy- Weinberg equilibrium and missingness), those with imputation INFO score <0.3 or MAF <0.01, or with missingness >0.1.

Replication

Replication of lead SNPs at loci associated with aortic traits was tested in a smaller independent dataset (SHIP). This was underpowered for discovery consistent effect direction is demonstrated in >89% of the overlapping lead SNPs for aortic dimensions, both in stage 1 GWAS and our MTAG analysis.
Direct replication of associations with distensibility was not possible due to lack of large enough dataset (distensibility data was not acquired as part of the SHIP study), but as distensibility is a phenotype derived from measures of aortic dimensions, the robustness of reported associations is supported by the replication data for aortic dimensions. Note that investigators of the only other large population study (MESA) that we are aware of for which MRI-derived distensibility data was obtained were unable to share their data with us at this time.
Replication of multivariate MR findings was attempted for aortic trait:WMH association in the only independent cohort for which we had access to both imaging-derived WMH and genotyping data (the Rhineland study). The analysis was underpowered with N=3317 (as compared with N>50,000 for the primary analysis using the CHARGE cohort); the conditional f-stat was very low (F<10) for the MVMR. We were therefore not able fully to replicate our primary findings. We noted that effect directions were consistent for the distensibility traits, but not for the area traits. The replication data is presented fully in the manuscript and Supplementary Tables.

Randomization	The main study design is a population study of genome-wide associations with aortic traits. No randomisation was required. However we did conduct Mendelian randomization analyses based on genetic associations in the study population (described in the Methods section) - here "randomisation" is by allele. We performed no other comparison between groups which would require randomization. We included covariates of sex, age, height, weight and genetic principal components in our association analyses.
Blinding	Analysts were not blinded because linking the genotype and phenotype data was necessary for quality control and analyses. Blinding was not relevant to our study. The participants from the included studies were sampled by multiple different research centers. The replications were conducted centrally on summary level results from each study.

Reporting for specific materials, systems and methods

We require information from authors about some types of materials, experimental systems and methods used in many studies. Here, indicate whether each material, system or method listed is relevant to your study. If you are not sure if a list item applies to your research, read the appropriate section before selecting a response.

Materials & experimental systems

n/a	Involved in the study	
☒	☐	Antibodies
☒	☐	Eukaryotic cell lines
☒	☐	Palaeontology and archaeology
☒	☐	Animals and other organisms
☒	☐	Clinical data
☒	☐	Dual use research of concern

Methods

n/a	Involved in the study	
☒	☐	ChIP-seq
☒	☐	Flow cytometry
☒	☐	MRI-based neuroimaging
